# A Comprehensive Review on the Therapeutic Potential of *Curcuma longa* Linn. in Relation to its Major Active Constituent Curcumin

**DOI:** 10.3389/fphar.2022.820806

**Published:** 2022-03-25

**Authors:** Shivkanya Fuloria, Jyoti Mehta, Aditi Chandel, Mahendran Sekar, Nur Najihah Izzati Mat Rani, M. Yasmin Begum, Vetriselvan Subramaniyan, Kumarappan Chidambaram, Lakshmi Thangavelu, Rusli Nordin, Yuan Seng Wu, Kathiresan V. Sathasivam, Pei Teng Lum, Dhanalekshmi Unnikrishnan Meenakshi, Vinoth Kumarasamy, Abul Kalam Azad, Neeraj Kumar Fuloria

**Affiliations:** ^1^ Faculty of Pharmacy, AIMST University, Kedah, Malaysia; ^2^ Faculty of Applied Sciences and Biotechnology, Shoolini University of Biotechnology and Management Sciences, Solan, India; ^3^ Department of Pharmaceutical Chemistry, Faculty of Pharmacy and Health Sciences, Royal College of Medicine Perak, Universiti Kuala Lumpur, Ipoh, Malaysia; ^4^ Faculty of Pharmacy and Health Sciences, Royal College of Medicine Perak, Universiti Kuala Lumpur, Ipoh, Malaysia; ^5^ Department of Pharmaceutics, College of Pharmacy, King Khalid University, Abha, Saudi Arabia; ^6^ Faculty of Medicine, Bioscience and Nursing, MAHSA University, Selangor, Malaysia; ^7^ Department of Pharmacology, College of Pharmacy, King Khalid University, Abha, Saudi Arabia; ^8^ Center for Transdisciplinary Research, Department of Pharmacology, Saveetha Dental College and Hospital, Saveetha Institute of Medical and Technical Sciences, Saveetha University, Chennai, India; ^9^ Department of Biological Sciences and Centre for Virus and Vaccine Research, School of Medical and Life Sciences, Sunway University, Selangor, Malaysia; ^10^ Faculty of Applied Sciences, AIMST University, Kedah, Malaysia; ^11^ College of Pharmacy, National University of Science and Technology, Muscat, Oman; ^12^ Department of Preclinical Sciences, Faculty of Medicine and Health Sciences, Universiti Tunku Abdul Rahman, Perak, Malaysia

**Keywords:** *Curcuma longa*, curcumin, phytochemical, pharmacology, toxicology

## Abstract

*Curcuma longa* Linn. (*C. longa*), popularly known as turmeric, belongs to the Zingiberaceae family and has a long historical background of having healing properties against many diseases. In Unani and Ayurveda medicine, *C. longa* has been used for liver obstruction and jaundice, and has been applied externally for ulcers and inflammation. Additionally, it is employed in several other ailments such as cough, cold, dental issues, indigestion, skin infections, blood purification, asthma, piles, bronchitis, tumor, wounds, and hepatic disorders, and is used as an antiseptic. Curcumin, a major constituent of *C. longa*, is well known for its therapeutic potential in numerous disorders. However, there is a lack of literature on the therapeutic potential of *C. longa* in contrast to curcumin. Hence, the present review aimed to provide in-depth information by highlighting knowledge gaps in traditional and scientific evidence about *C. longa* in relation to curcumin. The relationship to one another in terms of biological action includes their antioxidant, anti-inflammatory, neuroprotective, anticancer, hepatoprotective, cardioprotective, immunomodulatory, antifertility, antimicrobial, antiallergic, antidermatophytic, and antidepressant properties. Furthermore, in-depth discussion of *C. longa* on its taxonomic categorization, traditional uses, botanical description, phytochemical ingredients, pharmacology, toxicity, and safety aspects in relation to its major compound curcumin is needed to explore the trends and perspectives for future research. Considering all of the promising evidence to date, there is still a lack of supportive evidence especially from clinical trials on the adjunct use of *C. longa* and curcumin. This prompts further preclinical and clinical investigations on curcumin.

## Graphical Abstract

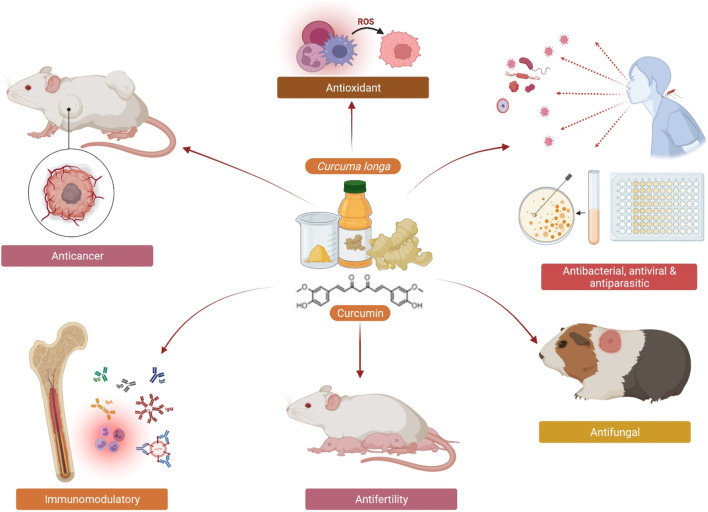

## Introduction

Herbs and natural products have long been exploited by humans to combat numerous diseases since the dawn of time. The Indian subcontinent boasts diverse flora, including both aromatic and therapeutic species. After all, contributions should be made to analyze, standardize, and confirm Unani and Ayurvedic medication for potential, safety, and effectiveness prior to actually introducing them to the market as first-line drug delivery. Plant-based therapies are being used across all civilizations. Plant-based medicines are already extensively utilized, and several countries invest 40%–50% of their total health budget to produce novel drugs. Herbal medicines are assumed to have a beneficial effect on health without any side effects.

The genus *Curcuma* has been employed from many years back due to its medicinal applications; it is composed of approximately 133 species worldwide. *C. longa* (Haridra), *C. aromatica* Salisb (Vana Haridra), *C. angustifolia* Roxb., *C. zanthorrhiza* Roxb., *C. amada* Roxb (Amaragandhi Haridra), *C. caesia* Roxb (Kali Haridra), and *C. zedoaria* Rosc (Zedoary) are common species of genus *Curcuma* found in several regions of the globe. *Curcuma longa* Linn. (*C. longa*) is the common tall herb that flourishes in tropical as well as in other Indian regions and is referred to as “Indian saffron or The Golden Spice of India” because of its use in a broad range of diseases in Indian homes as a spice, food preservative, and coloring source*. C. longa* belonging to the Zingiberaceae (ginger) family is a perennial plant commonly planted in Asian nations. It is among the oldest spices of India that have been used in Western and Southern parts for centuries, and is a significant part of Ayurvedic medicine. In Ayurveda, the therapeutic effects of *C. longa* have been well established and are discussed in Dashemani Lekhaniya (emaciating), Kusthagna (anti-dermatosis), and Visaghna (anti-poisonous) texts. It is known by many distinct names such as Haridra in Sanskrit, Haldi in Hindi, Jianghuang (yellow ginger in Chinese), manjal in South India, and Kyoo or Ukon in Japanese, which means an effective medication for jaundice (Sharma, 2000). *C. longa* is also extensively described in Indian material medica (Dravyaguna Shastra) and is used in the beauty regimen of Hindu girls where it is applied daily on their foreheads. The bride is smeared with a *C. longa* paste, which is a key aspect of Hindu tradition (Paranjpe and Pranjpe, 2001). It is well recognized by the Chinese, Japanese, and Korean Pharmacopoeias, and its application spans a broad range of medical conditions. In China, it is being used to relieve urticaria, dermatitis, hepatitis infection, inflammatory joints, sore throat, and wounds. It was mentioned in Hindu Mythology manuscripts as an aromatic stimulant and carminative. Turmeric powder combined with calcium hydroxide is indeed a popular home remedy for treating sprains and swelling induced by wounds or might be applied directly over the injury site. Traditional medicine has exploited dried curcumin powder to treat illnesses in history. *C. longa* is said to have antitoxic, anticancer, antibacterial, anti-inflammatory, and antioxidant effects (Ghotaslou et al., 2017). The tuberous rhizome from which *C. longa* is formed has a coarse and segmented skin. In the ground soil, the rhizomes mature underneath the foliage. The matured rhizomes have a yellowish-brown color with a dull orange from inside. Small pointed or tapered tubers sprout off the main rhizome measuring 2.5–7.0 cm (1–3 inches) in length and 2.5 cm (1 inch) in diameter (Prasad and Aggarwal, 2011). The dry rhizome is ground into a yellow powder form that has a bitter, yet sweet taste. A yellow-colored substance derived from the rhizome is curcumin (1,7-bis[4-hydroxy-3- methoxyphenyl]-1,6-heptadiene-3,5-dione), a combined form of resin and oil. Rhizome powder is supposed to flavor various cuisines and treat numerous disorders, including inflammation, flatulence, jaundice, menstrual troubles, hematuria, and hemorrhage. It is also a useful ointment to treat several skin disorders. Curcumin or diferuloylmethane and numerous volatile oils. *C. longa* of India is particularly popular when compared with those from other countries due to its high curcumin concentration, which is the most essential and active biological ingredient responsible for its therapeutic potential (Verma et al., 2018). Curcumin is a flavonoid having a lipophilic affinity that is practically water-insoluble (Dave et al., 2017) yet quite stable at the stomach’s acidic pH. *C. longa* and curcumin show antioxidant features close to vitamins C and E in both aqueous and fat-soluble extracts.

Due to shortcomings in the earlier published review articles, such as a lack of information on the therapeutic potential of *C. longa* in relation to its major compound curcumin, we have attempted to provide in-depth information by highlighting knowledge gaps in traditional and scientific evidence about *C. longa* in relation to the therapeutic potential of curcumin against numerous disorders. This review mainly focuses on the distribution, cultivation, botany, nutritional composition, phytochemistry, toxicology, traditional and medicinal properties, and safety aspects including the pharmacological activities of *C. longa* in relation to its major compound curcumin. This review will further discuss the current advances in *C. longa* and curcumin, such as the utilization of nanocarriers to increase curcumin bioavailability and overcome all the disadvantages in relation to drug delivery.

### Botanical Description, Geographical Distribution, and Cultivation of *C. longa*

**FIGURE 1:**
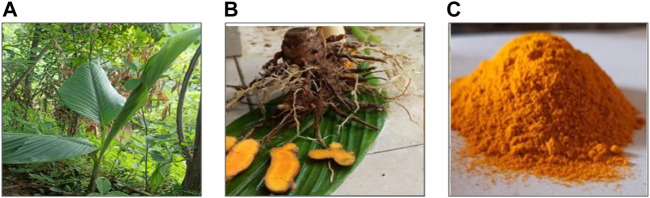
Important parts of *C. longa*. **(A)** *C. longa* in natural habitat, **(B)** medicinally important part of *C. longa* (rhizome), and **(C)** powder of dried rhizome of *C. longa* (used as a coloring agent in food).

**FIGURE 2:**
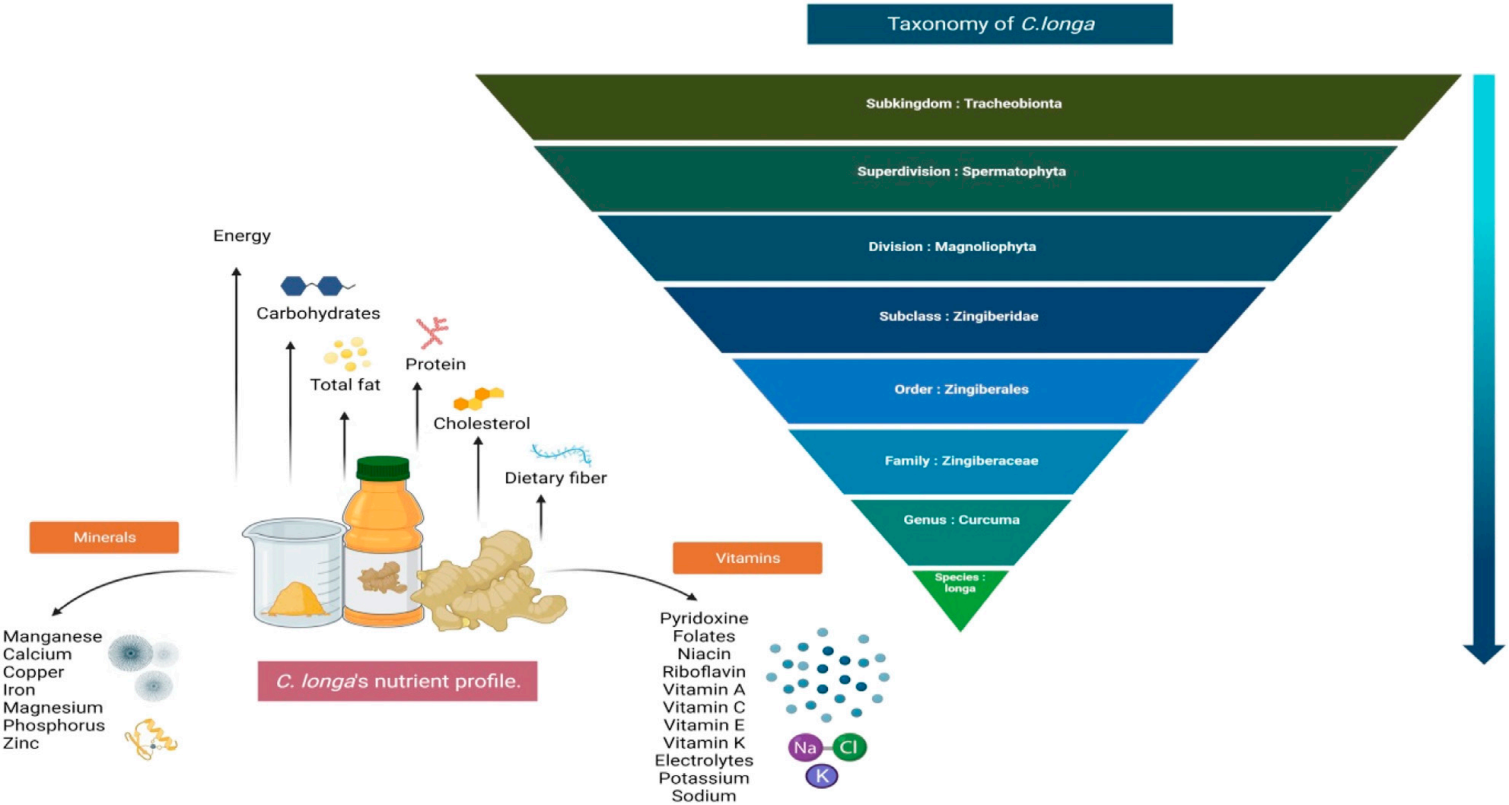
The taxonomic classification and nutritional profile of *C. longa.*

*C. longa* is a perennial herb with no stem and rootstock. Their leaves are 1 m long, lanceolate or oblong, dark green from the upper surface and pale green from beneath. The petiole and sheath are about the same length as the blade. Spike makes its appearance before the leaves. Flowers are sterile, pale yellow with a reddish covering, and flowering bract is green with a deep ferruginous purplish color. It has a 2-m-long, erect leafy shoot (pseudostems) (Rajkumari and Sanatombi, 2017) bearing 8–12 leaves and is commonly grown in rural backyard gardens. Its medicinally important parts are presented in Figure 1. The rhizomes have a balmy smell and bitter in taste (Puteri et al., 2020). The taxonomic classification of *C. longa* is shown in Figure 2.

Turmeric is believed to have originated from South or Southeast Asia, more likely in Vietnam, China, or western India. It is only identified as a domesticated plant and has not been found in the wild. India is the biggest producer, consumer, and supplier, but it is also cultivated extensively in Cambodia, Bangladesh, Nepal, Indonesia, Thailand, Cambodia, Malaysia, West Bengal, Madagascar, Tamil Nadu, Maharashtra, Madras Indonesia, and Philippines (Royal Botanic Gardens Kew, 2021). The turmeric plant requires an average temperature from 20 to 30°C and a good annual rainfall to grow. Plant species can reach 1 m in height and have long, oblong leaves. Turmeric can be found in both tropical and subtropical regions. This will thrive best in the dark if not overcrowded, but somehow it also develops larger and better rhizomes when exposed to sunlight. Turmeric usually grows in a humid environment. Harvest time usually lasts from January to March–April. Early types are ready in 7–8 months, while medium types mature in 8–9 months. After the formation of yellow colored leaves, they begin to dry and the crop is ready to be harvested (Soudamini and Kuttan, 1989). Upon ripening, cutting of the leaves are done nearer to the soil surface, the ground is ploughed, and the rhizomes are collected by hand picking or carefully lifting the clusters with a spade. The turmeric plant needs a rich and friable soil with just a little sand content. It grows in irrigated and rain-fed areas on light black, ashy loam, and red soils to stiff loams. Total irrigation for turmeric will be defined by the climatic and soil conditions. Based on the soil types and rainfall, 15–25 irrigations are provided in medium heavy soils, and 35–40 irrigations are needed in light texture red soils. Rhizomes are typically piled under trees for shade or in well-ventilated shelters and finally wrapping is done with turmeric leaves. Matured rhizomes as a seed could be kept in sawdust pits (Aggarwal et al., 2004).

### Traditional and Medicinal Properties of *C. longa*

*C. longa* rhizome is also consumed as an herbal infusion with conventionally produced gin called “ogogoro” by the native individuals of Delta State, Nigeria, with the notion that it heals various ailments. It is used to preserve and flavor food and is also used as a condiment in Nigeria. It is used for wound-healing and pimples in Pakistan and is widely consumed as folk medicine (Wahono et al., 2017). In Bhutanese traditional medicine, it is named Yung-ba, and it is employed as a tonic, an antidote, an antiseptic, an anti-inflammatory agent, and a preservative (Ayati et al., 2019).

*C. longa* is also developed in Thailand, Philippines, Sri Lanka, and Malaysia and is considered an ethnomedicinally important plant in Indonesia and Malaysia. Its poultice, when rubbed to the perineum, ensures the healing of any birth canal lesions. *C. longa* is also used to relieve dental issues and digestive troubles like discomfort or pain in the upper abdomen and acidity, indigestion, gas, and ulcers, as well as mitigate the hallucinogenic effects of hashish and other psychoactive drugs. The tribes of Jhalda, Parulia District of West Bengal, apply rhizome paste to the body to relieve physical pain. Assamese tribal women apply a fresh rhizome paste for skin infection and also to improve their complexion. Rhizome in addition to other ingredients cures loose stools in cattle. It is considered as a source of various problems such as blood purification, brain and heart tonic, asthma, leucoderma, piles, bronchitis, spleen enlargement, tumor, biliary disorders, anorexia, cough, rheumatism, sinusitis, tuberculous glands in the neck, diabetic wounds, hepatic disorders, leucorrhea, and gonorrheal secretion. It helps to lower blood clotting and blood sugar level (Zhang et al., 2013). Curcumin is now regarded as a promising “new medicine” that is being utilized as a supplement in a number of countries, namely, India, Japan, Thailand, Korea, China, Malaysia, and Pakistan; it is added to curry, tea, cosmetics, and drinks, and used as a colorant, antiseptic, and anti-inflammatory agent to treat gastrointestinal discomfort. It is also used as a component in cheese, butter, mustard sauce, and chips, and as a preservative and a dyeing agent in the United States. Curcumin is commercially available in multiple forms, including capsules, energy drinks, soaps, tablets, ointments, and cosmetics. Goud et al. (1993) found out that *C. longa* is the best source of ω-3 fatty acid and α-linolenic acid (2.5%).

**FIGURE 3:**
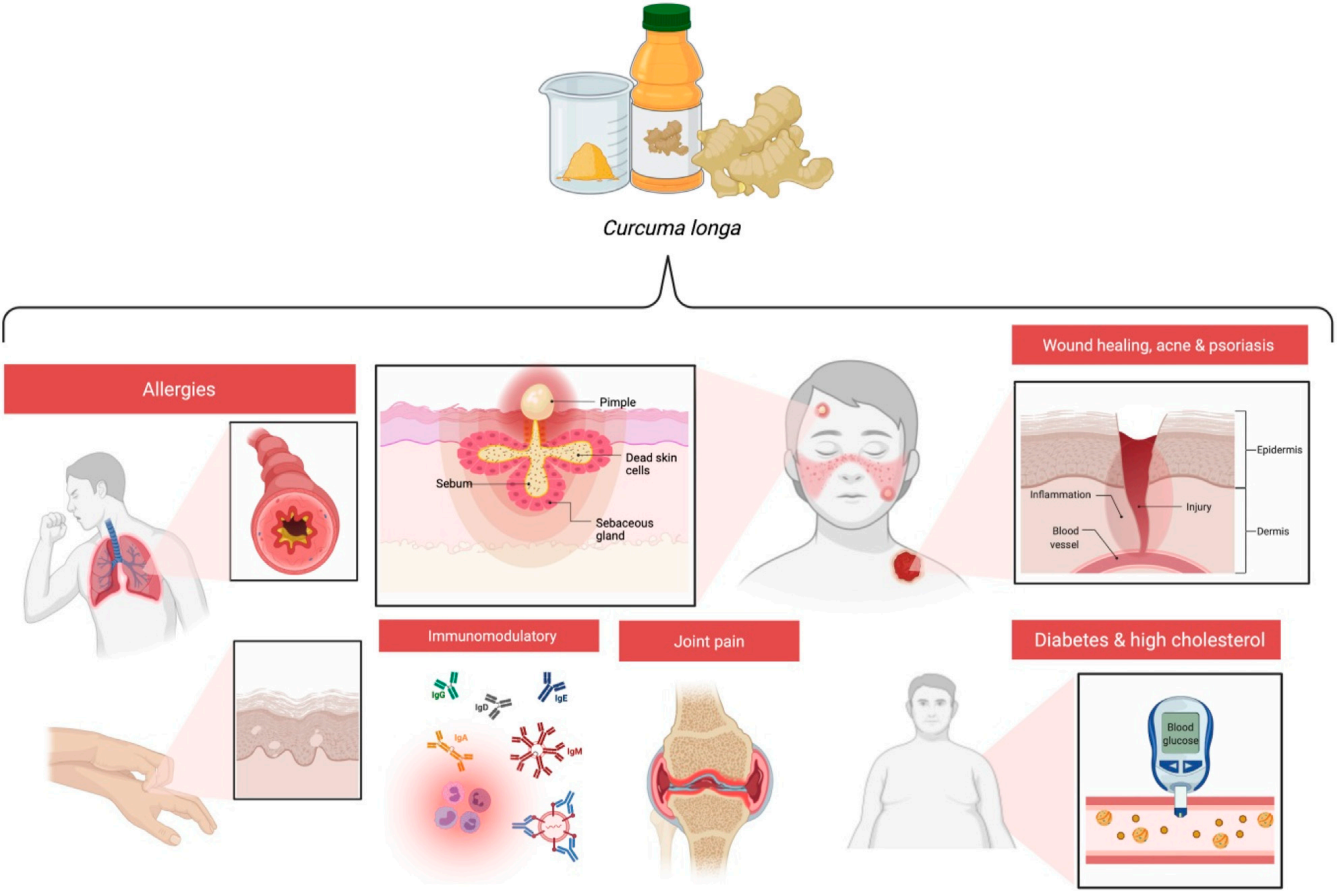
Benefits of curcumin in various conditions.

Rhizomes are being added to other plants to develop traditional remedies for a range of infections, such as tonsillitis, snake bites and stings, headaches, wounds, sprains, and fractured bones. Turmeric has been applied as a home remedy to heal wounds and also facilitates the treatment for digestive dysfunction, hepatic problems, leukemia, atherosclerosis, osteoarthritis, menstrual problems, bacterial infections, and eye problems. Turmeric has a role in preventing inflammation in the mucous membranes that line the throat, stomach, intestine, and lungs (Figure 3).

## General Health Benefits

Evidence suggests the benefits of turmeric in relieving acne, inflammation, joint pain, asthma, eczema, and tonic and acute allergies; in wound healing; in maintaining a balanced mood and blood sugar levels; and in immunomodulation (Ammon and Wahl, 1991; Reddy and Rao, 2002).

## Phytochemistry of *C. longa*

**FIGURE 4:**
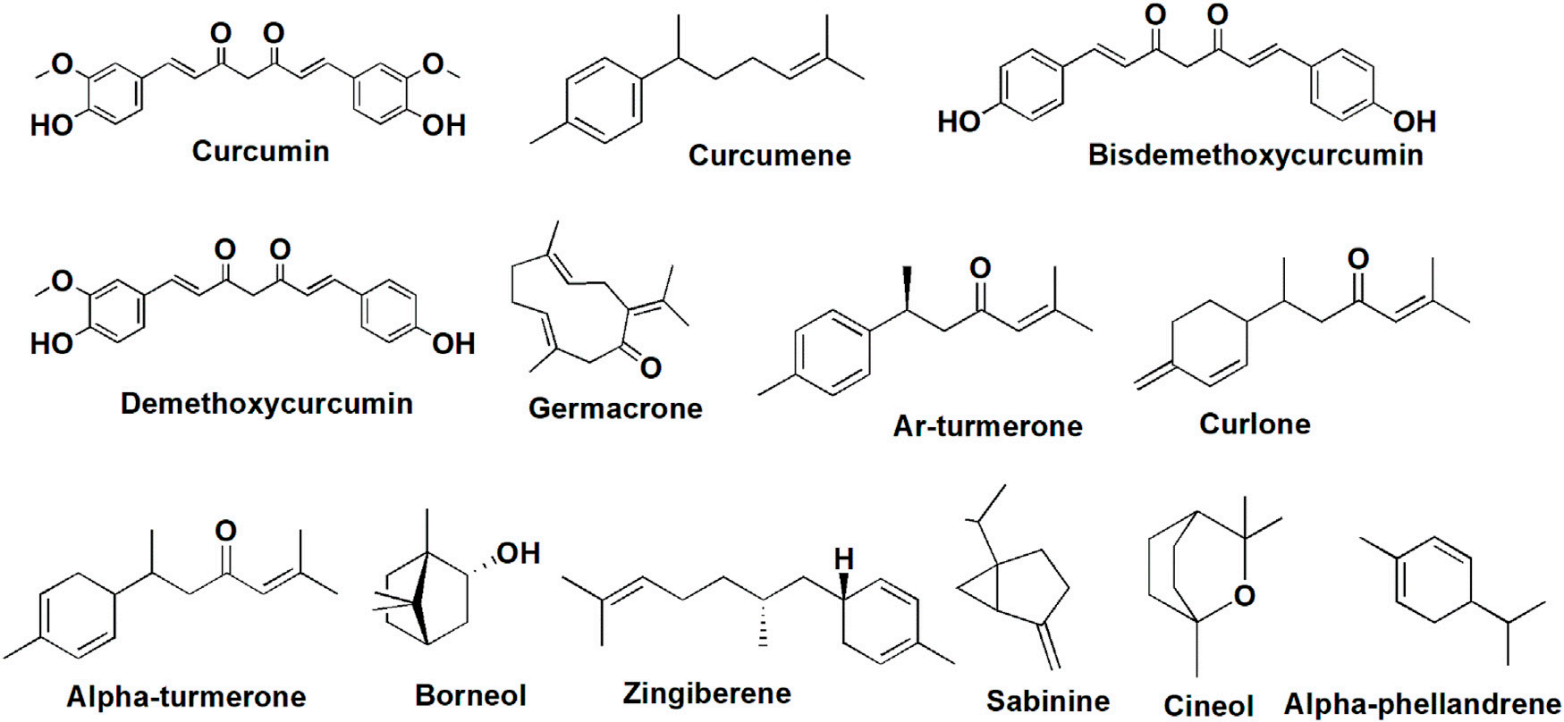
Chemical structure of phytoconstituents present in *C. longa.*

*C. longa* contains carbohydrates, fiber, certain proteins and lipids (no cholesterol), vitamin C, pyridoxine, magnesium, phosphorus, potassium, and calcium, which makes it a nutritionally rich natural food ingredient. The nutritional profile of *C. longa* is shown in Figure 2 (Pradeep et al., 1993). To date, 719 constituents have been isolated and recognized from 32 *Curcuma* species, including terpenoids, flavonoids, phenylpropene derivatives, alkaloids, diphenylalkanoids, steroids, and other compounds (Sun et al., 2017). The rhizome was found to contain over 235 phytoconstituents, the majority of which are polyphenols and terpenoids. Curcuminoids are made up of 80% curcumin and are the most common polyphenols. There are 109 sesquiterpenes, 68 monoterpenes, 22 diarylheptanoids and diarylpentanoids, eight phenolics, five diterpenes, four sterols, three triterpenoids, two alkaloids, and 14 remaining constituents. In a standard form, *C. longa* consists of moisture (>9%), curcumin (5–6.6%), extraneous matter (<0.5%), mold (<3%), and volatile oils (<3.5%). Monoterpenes dominate the essential oils of flowers and leaves, while sesquiterpenes dominate the oils of roots and rhizomes. The oil constituents contain 25% tumerone, 11.5% curdine, and 8.55% ar-turmerone (Nisar et al., 2015). *C. longa* oil contains anti-mutagenic qualities and is also capable of preventing the development and excretion of urinary mutagens in those who smoke cigarettes. According to the latest analysis, essential oil content in the rhizome was approximately 3.97%, with ar-turmerone (40%), α-turmerone (10%), and curlone (23%) being the major components analyzed by gas chromatography (Guimarães et al., 2020). The ar-turmerone has been employed as a repellant for insects, and its mosquitocidal ability has been revealed in the leaf extract. *C. longa* is a rich source of polyphenolic curcuminoids like curcumin (about 80%), demethoxycurcumin (about 12%), and bisdemethoxycurcumin (Ashraf, 2018), as well as proteins, volatile oils (tumerone, atlantone, and zingiberone), sugars, and resins. Curcumin, which makes up 0.3%–5.4% of raw *C. longa*, is the well-studied active ingredient. Table 1 illustrates the principal *C. longa* products, their appearance, chemical contents, and use. The *C. longa* plant is known to possess acidic polysaccharides (which include ukonan A, B, C, and D), 4.2% volatile oils (which include turmerone, ar-turmerone, curcumene, germacrone, and ar-curcumene as main constituents), and 5.8% essential oils (which include 0.6% sabinene, 0.5% borneol, 1% α-phellandrene, 1% cineole, 53% sesquiterpines, 25% zingiberene, and 3%–4% curcumin) (Chattopadhyay et al., 2004). Phenolic diketone curcumin provides yellow color, and consists of curcumin I (94%), curcumin II (6%), and curcumin III (0.3%). Protein (6.3%), fat (5.1%), minerals (3.5%), carbohydrates (69.4%), and moisture (13.1%) were reported by Liao et al. (2011). The main phytocompounds are presented in Figure 4.

**TABLE 1:** The main products of *C. longa*, their appearance, chemical constituents, and use.

Product name	Appearance	Chemical constituents	Uses
Whole rhizome (dried form)	Orange-brown, red-yellow, or pale yellow	3%–15% curcuminoids, and 1.5%–5% essential oils	Medicinal purposes
Ground *C. longa*	Yellow or red-yellow	Curcuminoids and essential oils may be reduced during the processing, as well as by light exposure. The powder must be stored in a UV-resistant container	Used as a condiment, dye, medicine, and dietary supplement
*C. longa* oil	Yellow to brown oil	Monoterpenes and sesquiterpenes are predominated in essential oils of leaves and rhizomes, respectively	Used as a spice, medicine, and dietary supplement
*C. longa* oleoresins	Dark yellow, reddish-brown viscous fluid	25% of essential oil and 37%–55% of curcuminoids	Used as a food dye, medicine, and dietary supplement
Curcumin	Yellow to orange-red colored crystalline powder	Curcumin and its bisdemethoxy and demethoxy derivatives. The three primary curcuminoids may account for up to 90% of the total curcuminoids. Oils and resins may make up a small percentage of the total composition.	Used as medicine and dietary supplement

## Pharmacological Properties of *C. longa*

*C. longa* claims to offer a wide range of therapeutic properties*. C. longa* was reported to contain curcuminoids, glycosides, terpenoids, and flavonoids. Haridra rhizome has been employed by healthcare professionals for diabetes, cholesterol, inflammation, diarrhea, liver problems, asthma, and cancer with minimal cytotoxicity to normal cells, and has been used as a cosmetic ingredient (Paranjpe and Pranjpe, 2001). In human trials, curcumin is suggested to be effective and safe, and the U.S. Food and Drug Administration has certified it as “generally regarded as safe”.

### Gastrointestinal Disorders

*C. longa* has long been used to treat digestive problems, and clinical investigations have verified its therapeutic advantages. Its anti-inflammatory action has been established in preclinical research to potentially protect the gastrointestinal tract. It has also been shown to increase gastrin, secretin, and bicarbonate secretion, as well as gastric wall mucus and pancreatic enzyme (Ammon and Wahl, 1991), as well as inhibit intestinal spasms and ulcer formation caused by stress, alcohol, indomethacin, pyloric ligation, and reserpine (Rafatullah et al., 1990) and improve dyspeptic patients’ condition. The activity of curcumin against inflammation and its therapeutic effect on gastrointestinal illnesses such as dyspepsia, *Helicobacter pylori* infection, Crohn’s disease, gastric ulcer, acidity, and ulcerative colitis in the form of fresh juice are thought to be antihelmintic. Curcumin inhibits nuclear factor (NF)-κB and reduces gastric mucosal damage in rats suffering from NSAID-induced gastropathy, leukocyte adhesions, intercellular adhesion molecule 1, and tumor necrosis factor (TNF)-α (Thong-Ngam et al., 2012). Between baseline and 8 weeks of treatment, *C. longa* tablet dramatically reduced irritable bowel syndrome (IBS) prevalence and abdominal pain/discomfort score, and IBS quality of life scores showed considerable improvement (Rahimi and Abdollahi, 2012). In male mice with liver damage, curcumin protects against acetaminophen-induced hepatitis by lowering oxidative stress and liver injury, and also restores glutathione levels (Somanawat et al., 2013).

### Respiratory Disorders

*C. longa* and its constituents have a relaxing impact on tracheal smooth muscles, suggesting a possible bronchodilatory influence in individuals with obstructive lung disease. They also have a protective benefit in an animal model of respiratory disorders, involving effects on inflammatory cells and mediators, lung pathological alterations, airway responsiveness, and immunomodulatory responses (Boskabady et al., 2020). Curcumin has been shown in both *in vivo* and *in vitro* investigations to have antiasthmatic properties. Curcumin therapy during OVA sensitization exhibited significant protective effects in an OVA-induced asthma paradigm in guinea pigs, attenuating bronchial constriction and hyperreactivity (Ram et al., 2003). Bronchitis is treated with fresh rhizome juice. *C. longa* is boiled in milk and combined with jiggery and used internally for rhinitis and cough. In cases of catarrhal cough and painful throat with infection, a rhizome decoction is gargled, and a piece of the rhizome is slightly burned and chewed. Turmerones, curcuminoids, curcumin, and tetrahydrocurcumin are chemical compounds of *C. longa* that have anti-asthmatic properties, and Haridradhumvarti (fumes wick) fumes are used in asthma and congestion.

### Inflammatory Disorders

**FIGURE 5:**
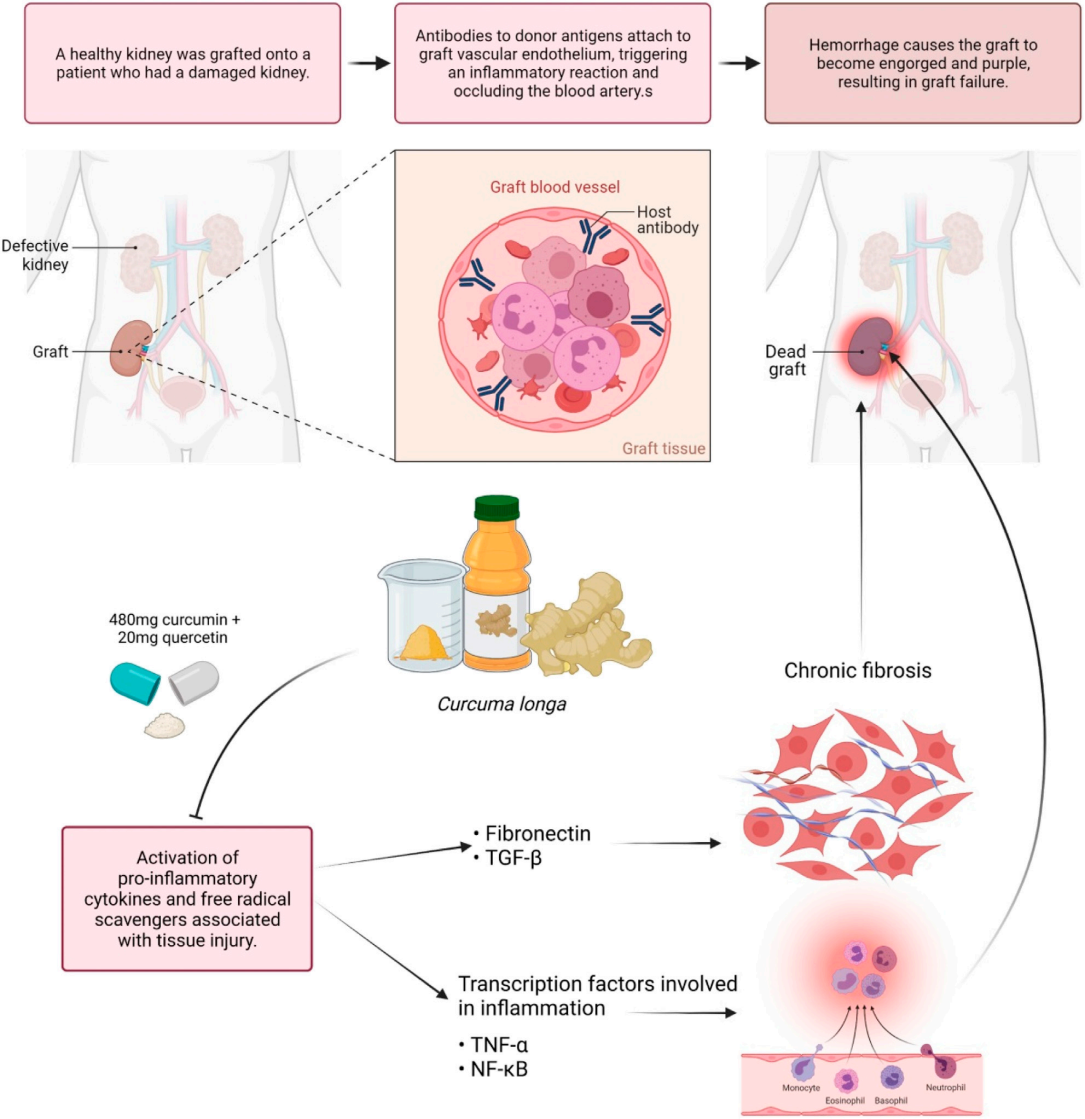
Modulation of antibodies by curcumin. Curcumin assists in the modulation of antibodies that react with endothelium and causes hyperacute graft rejection. Blocking the expression of pro-inflammatory cytokines and transcription factors linked to inflammation and fibrosis may help to prevent dead grafts.

Inflammatory markers include C-reactive protein (CRP), complements, and fibrinogen, all of which are induced by inflammatory cytokines in response to stimulation. According to Sandur et al. (2007), curcumin, demethoxycurcumin, and bisdemethoxycurcumin are the active compounds in *C. longa* that inhibit TNF-induced NF-κB activation. The methoxy groups on the phenyl ring were discovered to be responsible for their actions. *C. longa* extract was examined to improve serum inflammatory markers and mental health and mood disturbance in healthy participants who are overweight (Uchio et al., 2021). Researchers discovered that curcumin has anti-inflammatory properties by inhibiting the pro-inflammatory transcription factor (NF-κB) in 1995. They also discovered the molecular mechanism that underlies this inhibition (Singh and Aggarwal, 1995). TNF-α quickly activates NF-κB, which consists of the p50 and p65 subunits in human myeloid ML-1 cells, while curcumin prevented this activation. Curcumin also inhibits the binding of activator protein 1 (AP-1) binding factors, but the Sp1 binding factor remained unaffected. Curcumin inhibits the activation of NF-κB by phorbol ester and hydrogen peroxide, in addition to TNF-α. Furthermore, curcumin suppresses the NF-κB activation pathway after the convergence of multiple stimuli but before human I kappa B alpha phosphorylation. The capacity of *C. longa* to suppress both inflammatory prostaglandin derivative of arachidonic acid and neutrophil activity during inflammation may also indicate its anti-inflammatory activities. Curcumin is frequently used with bromelain to improve absorption and anti-inflammatory activity. Curcumin is equally efficacious as cortisone or phenylbutazone when given orally in acute inflammation*. C. longa* given orally to reduce inflammatory edema considerably. Curcumin’s therapeutic effect in sepsis appears to be achieved by activation of peroxisome proliferator-activated receptor gamma (PPAR-γ), which leads to inhibition of pro-inflammatory cytokine along with expression and release of TNF-α (Jacob et al., 2008). One trial evaluated 43 kidney transplant patients; 480 mg of curcumin and 20 mg of quercetin per capsule were observed to be potent during delayed graft rejection. Significant lower serum creatinine after transplant was attained in 43% of control patients and 71% of low-dose-treated participants. Induction of the hemeoxygenase enzyme, proinflammatory cytokines, and free radical scavenger associated with tissue injury possibly caused the enhanced early performance of transplanted kidneys (Figure 5) (Shoskes et al., 2006). Majority of the benefits seemed to be due to the anti-inflammatory and antioxidant properties of curcumin, while the quercetin in the molecule was negligible.

### Diabetes Mellitus

In diabetes mellitus, *C. longa* rhizome powder is particularly beneficial when added to amla juice and honey. Curcuminoids, the active component in the rhizome, reduce lipid peroxidation by keeping superoxide dismutase, catalase, and glutathione peroxidase active at higher levels. Curcuminoids have been demonstrated in diabetes mellitus type 2 patients to improve insulin resistance, reduce glucose and insulin levels, enhance adiponectin secretion, and lower levels of leptin, resistin, interleukin (IL-6, IL-1β), and TNF-α (Hajavi et al., 2017). According to the findings, *C. longa* ethanolic extract containing both curcuminoids and sesquiterpenoids is more hypoglycemic than curcuminoids or sesquiterpenoids alone (Nishiyama et al., 2005). *C. longa* extracts examined under *in vivo* conditions towards type 2 diabetes in mice models predict that it has a hypoglycemic impact by reducing blood glucose levels (Ponnusamy et al., 2012). *C. longa* has a low impact on postprandial plasma glucose and insulin in healthy individuals; it was found that consumption of 6 g of *C. longa* had no noticeable effect on the glycemic level. The change in insulin was substantially greater 30 and 60 min after the oral glucose tolerance test (OGTT) with *C. longa.* Following the OGTT, the insulin area under the curve was likewise considerably greater after consuming *C. longa.* Curcumin and its three derivatives (dimethoxy curcumin, bisdemethoxycurcumin, and diacetyl curcumin) were reported for their antioxidant capabilities (Faizal et al., 2009). *C. longa* dried rhizome powder diluted in milk has antidiabetic, hypolipidemic, and hepatoprotective properties, according to the scientific and systemic investigation, and could be used as an efficient and safe antidiabetic dietary supplement with great potential (Rai et al., 2010). Both isopropanol and acetone extract of *C. longa* inhibited human pancreatic amylase enzyme, which reduces starch hydrolysis, resulting in lower glucose levels (Ponnusamy et al., 2010).

### Cardiovascular Diseases

Cardiovascular diseases (CVDs) seem to be a global health issue that is linked to high disease and death rates. Anti-hypercholesterolemic, anti-atherosclerotic (Gao et al., 2019), and protective capabilities against cardiac ischemia and reperfusion (Wang et al., 2018) of curcumin have been proven in preclinical and clinical trials. Curcumin has anti-CVD potential by improving the lipid profile of patients, and it might be administered alone or as a dietary supplement to traditional CV medicines (Qin et al., 2017). Curcumin is also seen in many studies to protect against coronary heart disease (Li H. et al., 2020) and also possesses anticoagulant properties. CV preventive characteristics of *C. longa* include reduction in the level of cholesterol and triglycerides, decrease in the vulnerability of low-density lipoprotein (LDL) to lipid peroxidation, and platelet aggregation prevention, which helps to defend against atherosclerosis according to animal studies and also inhibits thromboxane formation. Curcumin increases VLDL cholesterol *trans*-protein plasma, causing increased levels and mobilization of α-tocopherol from adipose tissue that protects against oxidative stress that occurs during atherosclerosis. However, the fatty acids in the animals were less susceptible to oxidation in the blood vessel. It was suggested that oral intake of 500 mg/day curcumin for a week leads to a significant reduction in serum lipid peroxide (33%) and total serum cholesterol (12%) levels while increasing HDL cholesterol (29%). Curcumin may reduce chronic heart failure by boosting p38 MAPK, JNK, and ASK1, according to Cao et al. (2018). Curcumin and its components were used in recent research to determine the utility of nanotechnology-based medication delivery systems in CVD patients (Salehi et al., 2020).

### Hepatoprotective

In jaundice, rhizome powder added to amla juice is utilized. Jaundice is also cured by combining corriliyum (Anjana) with Haridra, Red ochre (Gairika), and Amalaki (*Emblica officinalis* Gaertn.) (Tripathi, 2009). *C. longa*’s hepatoprotective abilities have been proven in studies against several hepatotoxic ailments, including carbon tetrachloride, galactosamine, and acetaminophen (paracetamol) (Rao et al., 1995). The ethanolic crude extract of rhizomes was detected with curcumin, tumerone, atlantone, and zingiberene, which had substantial hepatoprotective ability at an oral dose of 250 and 500 mg/kg (Park et al., 2000). Karamalakova et al. (2019) investigated the decrease in the level of plasma bilirubin and gamma glutamyl transpeptidase, and the decrease in lipid peroxidation and provided significant hepatic protection against bleomycin toxicity by decreasing reactive oxygen species (ROS), which improves superoxide dismutase, catalase, and malondialdehyde levels. Curcumin is said to increase apoptosis in injured hepatocytes while also reducing inflammatory effects, hepatic fibrogenesis, and substantially liver injury. The hepatoprotective attribute of *C. longa* and curcumin might be due to direct free radical scavenging mechanisms, boosting glutathione levels, and assisting in liver detoxification. Aflatoxin-induced biliary hyperplasia, lipid alterations, and necrosis were likewise cured by *C. longa* and curcumin. Sodium curcuminate is a salt of curcumin that has choleretic effects, boosting biliary excretion of bile salts, cholesterol, bilirubin, and bile solubility, thus helping to prevent and treat cholelithiasis. This could be related to the antioxidant capacity of curcumin’s phenolic groups. Tacrine is well-known for its hepatotoxic and T-cell-destructive properties. Curcumin was over ten times more efficient than standard therapy, ascorbic acid, in research involving human hepatocytes cells that had been disrupted by tacrine (Song et al., 2001).

### Neuroprotective Activity

*Curcuma* oil lowers ischemia’s negative effect by decreasing nitrosative and oxidative stress. Ischemia collapses the membrane potential of the mitochondria, cytochrome c releases, Bax:Bcl-2 protein ratio changes, and caspase-activated, which leads to the apoptotic initiation in a sequential manner, which was considerably inhibited by *Curcuma* oil. As a result, there is evidence for the action of *Curcuma* oil in neuroprotection with a wide therapeutic window for the reduction in ischemic brain injury (Dohare et al., 2008).

In an Alzheimer’s disease transgenic mouse, curcumin decreased oxidative stress and repaired amyloid pathology. Antioxidant and anti-inflammatory features of curcumin helped to minimize the manifestation of Alzheimer’s disease, which is characterized by inflammation and oxidation. Parkinson’s disease (PD) is found to be the second most common neurodegenerative disease following Alzheimer’s disease, which affects dopaminergic neurons of the substantia nigra pars compacta (SNpc) and decreases dopamine (DA) in their striatal terminals. Curcumin is suggested to be an effective therapeutic and nutraceutical agent for PD treatment. Interestingly, curcumin was found to inhibit the synthesis of MOA-B enzyme (Khatri and Juvekar, 2016), which would lead to an increase in the level and availability of DA in the brain. Neuroprotective effects of curcumin in a 6-hydroxydopmine animal model of PD (El Nebrisi et al., 2020) indicated an increase in the survival of striatal TH fibers and SNpc neurons, decreased abnormal turning behavior, and exerted neuroprotective properties. These findings provide evidence that α7-nicotinic acetylcholine receptors could be a potential therapeutic target and curcumin would be the first natural source that is found to modulate nicotinic receptors in PD. Curcumin can be a future therapy for various neurological illnesses including major depression, involuntary movement, as well as diabetic neuropathy (Kulkarni and Dhir, 2010). Ethanol extract of *C. longa* was found to show neuroprotective effects on neuronal loss induced by dexamethasone treatment in rat hippocampus (Issuriya et al., 2014). In 2018, an *in vivo* study revealed that administration of *C. longa* extract at a dose of 200 mg/kg in trimethyltin (TMT)-treated Sprague–Dawley rats with neurotoxic damage seems to prevent the deficits in the spatial memory performance and partially inhibit the decrease in the number of CA2–CA3 region pyramidal neurons. Therefore, the anti-inflammatory as well as antioxidant effects of *C. longa* were observed (Yuliani and Mustofa, 2018) Furthermore, Yuliani and Mustofa (2019) examined the neuroprotective effects of ethanolic *C. longa* extract at 200 mg/kg in an *in vivo* analysis *via* preventing oxidative stress by decreasing the plasma and brain malondialdehyde levels and increasing the superoxide dismutase, catalase, and glutathione peroxidase enzyme activities and glutathione levels in the brain on TMT-exposed Sprague–Dawley rats. Terrestrial animals and aquatic animals are also required to be used for research purposes. An aquatic environment serves as a sink for environmental contaminants including Benzo[a]pyrene (B[a]P), and research on the fish model is also needed to understand the influence of B[a]P on oxidative stress-induced neurotoxicity and anxiety-like behavioral responses in aquatic animals (Billiard et al., 2006; Satpathy L. and Parida S. P., 2021). B[a]P is important in the mechanical aspects of oxidative stress to lipid membranes, nucleic acids, and proteins, as well as changes in antioxidant capacity. Curcumin has a potential to act as a co-supplement by reducing anti-anxiety behavioral response and altering antioxidant activity with a significant increase in pyknotic neuronal counts in the periventricular gray zone of the optic tectum that regulates anxiety against B[a]P-induced neurotoxicity in adult zebrafish (Satpathy L. and Parida S., 2021).

Banji et al. (2021) demonstrated the neuroprotective and antioxidant activity of *C. longa* extract in synergy with essential oil against neurotoxicity mediated by aluminum. Detection of free curcumin and its metabolites in the brain and plasma has increased bioavailability and tissue distribution, implying that it could be used in neurodegenerative illnesses.

Another neurodegenerative disease, amyotrophic lateral sclerosis (ALS), causes a selective loss of motor neurons in the spinal cord, brainstem, and motor cortex. Curcumin was studied to determine if it could help ALS patients, particularly those with bulbar involvement, survive longer (Ahmadi et al., 2018). Curcumin therapy reduced the development of ALS and oxidative damage in a double-blind therapeutic trial (Chico et al., 2018). Curcumin-based drug delivery systems are beneficial for the treatment of ALS, according to a study (Tripodo et al., 2015), although Rakotoarisoa and co-workers pointed out that curcumin has chemical instability, low oral bioavailability, and low water solubility rate in the ALS disease condition (Rakotoarisoa and Angelova, 2018).

The ability of curcumin to interact indirectly with a diverse array of transcription factors, including NF-κB, activator protein 1 (AP-1), β-catenin, and signal transducer and activator of transcription (STAT) proteins, and to act as a partial agonist of the PPAR-γ, a ligand-activated transcription factor involved in both neuroprotective and anti-inflammatory signaling pathways (Chen et al., 2015; Kunnumakkara et al., 2017). Curcumin has been demonstrated to help with a variety of diseases, including multiple sclerosis (MS) (Mohajeri et al., 2015). Curcumin-D-monoglucuronide (curcumin monoglucuronide, CMG) was developed as a prodrug form of curcumin due to its low bioavailability in the body. CMG is deemed to be safe for use and can be injected intravenously, revealing an anticancer impact on mice implanted with human colorectal cancer cells by achieving a 1,000-fold higher blood concentration of free-form curcumin than curcumin administered orally (Ozawa et al., 2017). In mouse xenograft models, CMG given intraperitoneally appears to have antitumor effects on oxaliplatin-resistant colon cancer with minimal toxicity (Ozawa-Umeta et al., 2020). After CMG delivery, the microbiota changes, which may be linked to immunopathology suppression in an autoimmune model for MS (Chearwae and Bright, 2008) and experimental autoimmune encephalomyelitis (EAE). The gut microbiota has been suggested to play a major role in the development and severity of MS. When compared to healthy controls, MS patients had an increased number of bacteria from the genera *Akkermansia, Blautia*, and *Pseudomonas*, as well as a lower number of bacteria from the genera *Prevotella* and *Parabacteroides* (Chen et al., 2016; Park et al., 2017; Tsunoda, 2017).

### Antioxidant Properties

*C. longa* and its curcumin constituent have significant antioxidant activity, equivalent to both vitamin C and vitamin E, in both water- and fat-soluble extracts. Curcumin can help the body rid itself of hydroxyl radicals, singlet oxygen, superoxide radicals, nitrogen dioxide, and NO. Curcumin pretreatment was proven to reduce ischemia-induced mutations in the heart (Dikshit et al., 1995). The efficiency of curcumin on endothelial heme oxygenase-1 (inducible stress protein) employing bovine aortic endothelial cells was discovered in an *in vitro* investigation that resulted in increased cellular resistance to oxidative stress. Curcumin can also help *Caenorhabditis elegans* live longer by lowering intracellular ROS and lipofuscin levels during aging (Liao et al., 2011). Previous research into the potential of *C. longa* to sustain hippocampal cells of male Wistar rats from lead-induced damage and reduces lipid peroxidation caused by toxic heavy metals. Resveratrol and curcumin alleviate and synergistically repair oxidative stress to the tissues by enhancing antioxidant response through free radical scavenging (Al-Basher et al., 2020). In one of the earlier studies, the anti-inflammatory and antioxidant capability of curcumin was detected to be synergistically enhanced with quercetin, and a synergistic protective effect was also demonstrated in diazinon-induced rats (Abdel-Diam et al., 2019). The anti-inflammatory impact of berberine and curcumin may decrease oxidative stress, liver inflammation, and lipid metabolism (Feng et al., 2018), and the berberine combination also reduced inflammatory and oxidative stress responses in the cortex and hippocampus of rats (Lin et al., 2020).

### Anticancer Activity

**FIGURE 6:**
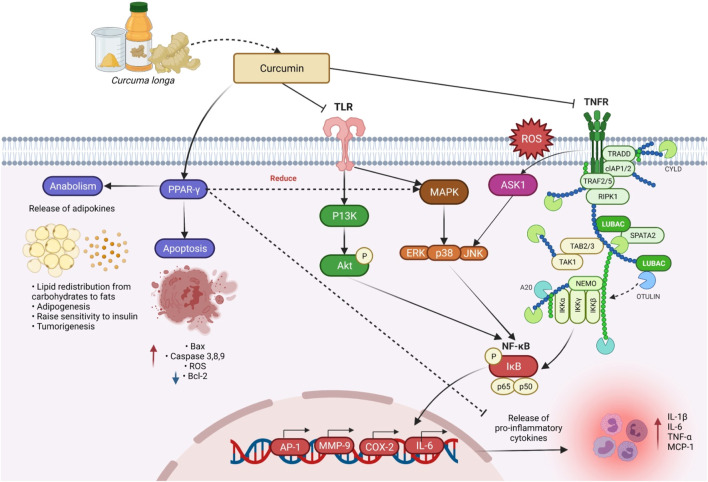
Curcumin’s mechanism of action in reducing inflammation, anabolism, and apoptosis. By inhibiting the pro-inflammatory transcription factor (NF-κB), and activation of PPAR-γ, curcumin aids in anabolism and apoptosis, suppression of pro-inflammatory cytokines, as well as the expression and release of TNF-α. Abbreviations: TLR, Toll-like receptors; TNFR, Tumor necrosis factor receptor; ROS, Reactive oxygen species; TRADD, Tumor necrosis factor receptor type 1-associated death domain protein; CYLD, CYLD lysine 63 deubiquitinase; cIAP1/2, Cellular inhibitor of apoptosis protein 1/2; TRAF 2/5, Tumor necrosis factor receptor-associated factor 2/5; RIPK1, Receptor-interacting serine/threonine-protein kinase 1; LUBAC, Linear ubiquitin chain assembly complex; SPATA2, Spermatogenesis-associated protein 2; NEMO, NF-κB essential modulator; TAB2/3, TGF-beta activated kinase 1 (MAP3K7) binding protein 2; TAK1, Transforming growth factor-β-activated kinase 1; IKKα, IKKβ, and IKKγ, Inhibitory kappa b kinase alpha, beta, and gamma; IkB, Inhibitor of nuclear factor kappa Bv; PPAR-γ, Peroxisome proliferator-activated receptor gamma; P13K, Phosphoinositide 3-kinases; Akt, Ak strain transforming; ERK, Extracellular-signal-regulated kinase; JNK, Jun N-terminal kinase; Bax, Bcl-2-associated X-protein; AP-1, Activated protein-1; MMP-9, Matrix metallopeptidase 9; COX-2, Cyclooxygenase 2; IL-6 and 1β, Interleukin 6 and 1 beta; TNF-α, Tumor necrosis factor alpha; MCP-1, Monocyte chemoattractant protein-1.

Annapurna et al. (2011) evaluated the ability of *C. longa* prophylactically and therapeutically, i.e., pre-induction treatment and post-induction treatment *via* oral and topical application to modulate the N-methyl-N-nitrosourea-induced mammary cancer in rats for 24 weeks. Prophylactic topical application given at 200 mg/kg of *C. longa* has significantly reduced the mean tumor volume compared with therapeutic topical application. This was the first report to show the anticancer activity of *C. longa* with topical application in a breast cancer model. In an *in vivo* research involving the topical application of curcumin in CD-1 mice and dietary administration of 1% *C. longa*, 0.05% of its ethanol extract significantly reduced tumor incidence, tumor burden, and tumor volume in dimethyl benz[a]anthracene (DMBA)-initiated and 12,O-tetradecanoylphorbal-13-acetate (TPA)-promoted skin tumors (Huang et al., 1988). Kuttan and his colleague’s work was the first to demonstrate curcumin’s anti-cancer potential in both *in vitro* and *in vivo* experimental models (Kuttan et al., 1985). Curcumin activates DNA damage response, laying the foundation for the therapeutic use of these nutraceuticals in prostate cancer chemoprevention (Horie, 2012). The general anti-carcinogenic effect of curcumin involves mechanisms like induction of apoptosis and inhibition of cell-cycle progression in rat aortic smooth muscle cells (Chen and Huang, 1998). The antiproliferative effect is regulated partly through hindrance of protein tyrosine kinase activity and c-myc mRNA expression, while the apoptotic effect may partly be mediated *via* preventing the functioning of protein tyrosine kinase, protein kinase C, and expressions of c-myc mRNA and bcl-2 mRNA (Chen and Huang, 1998). Curcumin inhibits the transcription factor NF-*κ*B (Figure 6) and various downstream gene products like c-myc, Bcl-2, COX-2, nitric oxide synthase (NOS), Cyclin D1, TNF-α, ILs, and matrix metallopeptidase 9 (MMP-9) and has anti-proliferative activities in a diversity of malignancies. Curcumin could be used to avoid colorectal cancer (CRC) in diabetics with type 2 diabetes by lowering leptin blood levels and increasing adiponectin levels. Poloxamer 407 can be employed as a polymer to expand the colorectal medicine liberation mechanism for curcuminoids in CRC treatment, according to the study of Chen et al. (2012).

A novel approach in adjuvant treatment for osteosarcoma is by combining a synthetic counterpart of the natural chemical pancratistatin with curcumin (Ma et al., 2011). One controlled study found that employing poly-lactic-co-glycolic acid to create and characterize nano-curcumin improves the water solubility as well as anticancer activity of the nanoparticulate emulsion (Nair et al., 2012). Curcumin also has an impact on numerous growth factor receptors and adhesion molecules that are implicated in tumor growth, angiogenesis, and metastasis (Wilken et al., 2011) and exerts antitumor action in cancer cells by the suppression of NF-*κ*B and signal transducers and activators of the transcription 3 (STAT3) pathways (Jiménez-Flores et al., 2014).

A study on *in vitro* and *in vivo* models revealed that both *C. longa* and curcumin exhibited the ability to lessen the impacts of numerous known causative agents of mutation and cancer in different body tissues. Curcumin (50 μM) initiates destruction in the human kidney cells and causes the colorectal HT-29 cancerous cells to grow larger, which is most probably due to cell cycle arrest (Kössler et al., 2012). Curcumin also triggers programmed cell death in colon cancerous cells and inhibits micro-inflammation in the gastrointestinal system linked to inflammatory bowel illnesses, according to laboratory research (Nita, 2003). Okanlawon et al. (2020) determine the influence of the inclusion of powdered *C. longa* on carcass yield and intestinal increase in rabbit production. Farombi et al. (2007) explored the combined effects of curcumin and kolaviron (a bioflavonoid extracted from Garcinia kola seeds) on DBP-induced testicular injury in rats. Curcumin treatment of mice infected with human prostate cancer cells resulted in a lowered microvessel density, cell proliferation, an improvement in apoptosis. Endothelial cells derived from bovine aorta exposed to curcumin (5–15 μM) under normoxic (oxygen tensions within 10–21%) or hypoxic (oxygen tensions within 1–5%) conditions were reported to increase heme oxygenase activity and resistance to oxidative stress. Consumption of alcohol sensitizes the pancreas to give an inflammatory response through NF-κB activation *via* protein kinase C epsilon. One pilot study concluded that an oral dosage of 500 mg of curcumin with 5 mg of piperine could restore lipid peroxidation in patients suffering from tropical pancreatitis (Durgaprasad et al., 2005).

EGFR-, miRNA-, autophagy-, and cancer stem cell-based treatments with curcumin could be proven as potential processes and targets for tackling lung cancer (Ye et al., 2012). Curcumin also seems to promote tumor progression, reducing the efficiency of docetaxel in lung cancer patients. Meanwhile, synchronized curcumin and docetaxel treatment causes minor toxicity in normal organs, as well as the bone marrow and liver (Yin et al., 2012). *In vivo* curcumin lessens the migratory and invasive capabilities of A549 cells and inhibited adiponectin expression thought to be mediated through the NF-κB/MMP pathways and has been proposed as an adjuvant in lung malignancy (Tsai et al., 2015).

**FIGURE 7:**
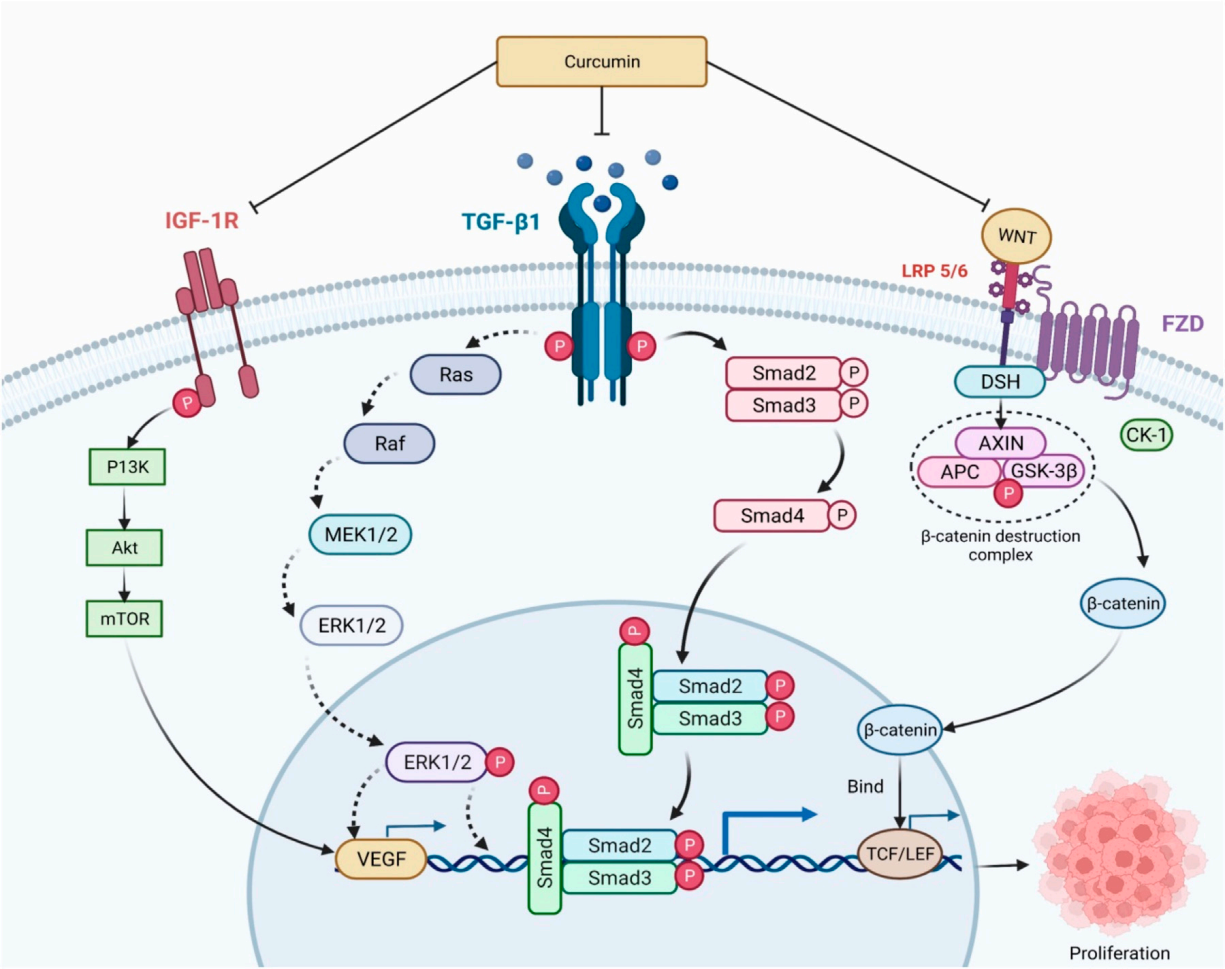
Mechanism of curcumin in regulation of cancer proliferation. TGF-β1/smad3, IGF, PI3K/Akt, Wnt/β-catenin, and vascular endothelial growth factor (VEGF) are some of the signaling pathways and molecular targets that curcumin modulates to inhibit cancer. Blocking these receptors has the potential to reduce chronic inflammation and oxidative damage. DSH and AXIN are recruited once the WNT binds to LRP 5/6, producing the β-catenin destruction complex. β-catenin that has escaped into the nucleus promotes the transcription of genes including cyclin D1 and P13k, which promote cell proliferation and growth. IGF-1R, Insulin-like growth factor 1 receptor; TGF-β1, Transforming growth factor beta 1; WNT, Wingless/integrated; LRP 5/6, Low-density lipoprotein receptor-related protein 5/6; Smad, small mothers against decapentaplegic; Ras, Rat sarcoma virus; Raf, Rapidly Accelerated Fibrosarcoma; MEK 1/2, Mitogen-activated protein kinase 1/2; ERK ½, Extracellular-signal-regulated kinase ½; P13K, Phosphoinositide 3-kinases; Akt, Ak strain transforming; mTOR, mammalian target of rapamycin; TCF/LEF, T-cell factor/lymphoid enhancer factor; FZD, Frizzled; DSH, Dishevelled; AXIN, Axis Inhibitor; APC, Adenomatous polyposis coli; GSK-3β, Glycogen synthase kinase-3 beta; CK-1, casein kinase 1.

Yu and his colleagues evaluated the role of curcumin in inhibiting the human hepatoma SMMC-7721 cells significantly by promoting apoptosis *via* modulation of Bax/bcl-2 (Yu et al., 2011). Apoptosis was associated with increases in p53 levels as well as its DNA-binding ability, along with protein expression of Bax. Phosphorylation of CDC27 (cell division cycle 27) is the main mechanism of anticancer efficacy of curcumin by obstructing cell growth and proliferation in an apoptotic pathway, leading to the death of the cells (Lee and Langhans, 2012). It has been discovered that circulating miR-21 is elevated in patients with hepatocellular carcinoma (HCC); it can be exploited as a diagnostic marker and therapeutic target for HCC, and is being linked to distant metastasis (Zhang et al., 2019). According to Li and his colleagues, in human hepatoma cell lines such as HepG2 and HCCLM3, suppression of miR-21 improved anticancer action of curcumin like cell growth suppression, apoptosis *via* upregulated target gene, and TIMP3 expression, and the mechanism may refer to TGF-*β*1/smad3 signaling pathway inhibition (Li J. et al., 2020). Curcumin inhibits cancer through modulating several signaling pathways and molecular targets, including TGF-β1/smad3, IGF, PI3K/Akt, Wnt/β-catenin, and vascular endothelial growth fact (VEGF) (Figure 7) (Mohebbati et al., 2017). Although several reasons for curcumin’s antitumor potential have been hypothesized, the exact molecular mechanism of this activity against HCC is unknown.

Curcumin therapy of Burkitt’s lymphoma cell lines in combination with ionizing radiation shows that it boosts lymphoma cells’ susceptibility to ionizing radiation-induced apoptosis and improves cell cycle arrest at the G2/M phase. Curcumin and L-ASP show synergism in patients with blood and bone marrow malignancy (Jiang et al., 2015). Curcumin also hinders the cellular growth of uterine leiomyosarcoma and reduces the spread of castrate-resistant disease and human leiomyosarcoma cells *via* modulating the AKT-mammalian target of rapamycin pathway for inhibition (Wong et al., 2011). Curcumin or *C. longa* extract’s potential in decreasing tumors induced chemically was investigated. It was documented that curcumin and crude extract of *C. longa* is useful in reducing papilloma development throughout carcinogenesis and progression. About 0.2% and 1.0% of dietary curcumin can reduce the number of papilloma by acting on 7,12-dimethyl benz[a]anthracene (DMBA) and 12,0-tetradecanoylphorbol-13-acetate (TPA) that promoted skin tumor, which was explored by Limtrakul et al. (2001) as ras-p21 and fos-p62 oncogene expression was decreased dose-dependently by curcumin. Mohanty et al. (2006) examined the potency of *C. longa* on apoptosis of myocardial cells in experimentally produced myocardial ischemic–reperfusion injury because it exhibited considerable anti-apoptotic effects that lead to the preservation of cardioprotective characteristics and heart function. Aqueous *C. longa* extract exhibited antimutagenic activity against mutagens and also inhibited the progression of forestomach tumors induced by benzo[a]pyrene against *Salmonella typhimurium* strains.

Insulin-like growth factor-binding protein-3 (IGFBP-3) is a high-affinity binding protein that alters the mitogenic functions of IGFs while also having anti-proliferative and proapoptotic characteristics. Apoptosis is induced by transfection of IGFBP-3 cDNA into breast cancer cell lines expressing either mutant (T47D) or wild-type p53 (MCF-7). IGFBP-3 results in a higher ratio of pro-apoptotic to anti-apoptotic Bcl-2 family members. Curcumin induced cell apoptosis in MCF-7 *via* a p53-dependent pathway and could offer therapeutic promise in patients with breast cancer (Choudhuri et al., 2002). In the mouse model, the combination of curcumin and cyclophosphamide negated the efficacy of cyclophosphamide and then hindered the reduction of tumor size. Curcumin causes DNA fragmentation and base degradation in the presence of copper and cytochrome p450 isoenzymes. Furthermore, Frank et al. (2003) demonstrated that curcumin bonded to copper did not suppress spontaneous hepatic tumor growth in a rat model of liver cancer. The enhanced toxicity and oxidative stress may be explained by the excess load of copper.

Curcumin can limit the absorption and effectiveness of irinotecan, a chemotherapy medication used in colon cancer. Curcumin in combination with paclitaxel (Taxol) effectively decreased breast cancer dissemination to the lung in a xenograft mouse model of human breast cancer, relative to either curcumin or paclitaxel alone (Frank et al., 2003). Curcumin reduces T cells significantly, but a low dosage of curcumin boosts T cells retrieved from mice with the 3LL tumor. Consequently, increased CD8^+^ T cells demonstrated improved IFN-*γ* secretion and proliferation, especially against 3LL tumor cells (Han et al., 2014). *C. longa* extracts (containing curcuminoids, volatile oil, and water-soluble polysaccharides) could be employed as an adjuvant supplement for cancer patients who have their immune systems impaired by chemotherapy and saw an improvement in the expansion of peripheral blood mononuclear cells and the composition of cytokines. *In vitro* and *in vivo* analyses have indicated that aromatic-turmerone exhibits anti-angiogenic abilities on human endothelial cells (Yue et al., 2015).

Dietary substances such as curcumin and docosahexaenoic acid (DHA) have varying antiproliferative abilities among multiple breast cell lines and indicated synergism in SK-BR-3 (human breast cancer cell line) cells, which might be due to DHA elevation of cellular curcumin absorption and, thus, an occurrence unique to the combined action of substances (Altenburg et al., 2011). The study shows the synergistic interaction between curcumin and garcinol in pancreatic cancer cells (BxPC-3 and Panc-1) in a dose-dependent manner (Parasramka and Gupta, 2012). *In vitro* screening is carried out on human multiple myeloma cell lines (U266) by Ghoneum and Gollapudi (2011) to explain the synergistic apoptotic potency of arabinoxylan rice bran (MGN-3/Biobran) and curcumin (*C. longa*) in the United States.

### Anti-Allergic Activity

Curcumin inhibited the degranulation and release of histamine from rat peritoneal mast cells caused by compound 48/80. Calcium uptake measurements and cAMP tests in mast cells were used to investigate the mechanism of action. In an animal model, curcumin dramatically reduced the mast cell-mediated passive cutaneous anaphylactoid reaction. Curcumin enhanced intracellular cAMP levels and inhibited both nonspecific and selective mast cell-mediated allergy reactions (Choi et al., 2010). Curcumin significantly reduced IgE/Ag-induced PSA (passive systemic anaphylaxis), as measured by serum-dependent leukotriene C4, dependent prostaglandin D2, and histamine levels, indicating that it might be useful to produce drugs for allergic inflammatory illnesses (Li et al., 2014). Curcumin can suppress expression of CD80, CD86, and class II antigens by dendritic cells and blocks the release of inflammatory cytokines like IL1β, IL-6, and TNF-α from LPS-stimulated dendritic cells.

### Antidermatophytic Activity

Rhizome juice is utilized as an antiparasitic in the therapeutics of numerous skin problems, and rhizome powder is added to cow’s urine to relieve internal itching and dermatitis (Paranjpe and Pranjpe, 2001). Leaves hold great potential against human pathogenic fungi on account of their various *in vitro* and *in vivo* antifungal activities, for instance, strong fungicidal ability, long shelf life, high inoculum density tolerability, thermostability, a large number of antidermatophytic effects, and lack of any side effects. Curcumin has been proven to have antimutagenic, antioxidant, free radical scavenging, anti-inflammatory, and anti-carcinogenic abilities, allowing it to protect the skin from detrimental UV-induced impacts (Binic et al., 2013).

### Pregnancy/Neonates

*C. longa* and curcumin caused a considerable increase in hepatic glutathione S-transferase (GST) and sulfhydryl (SH), Cytochrome b5, and cytochrome P450 levels, implying that *C. longa* and/or curcumin metabolites can be passed down through the milk supply. *C. longa* and curcumin are nontoxic and non-mutagenic during pregnancy in animals, although additional research in humans is needed (Soleimani et al., 2018).

### Irritable Bowel Syndrome

IBS patients have far more inflammatory cells in the mucosa of the colon and ileum part of the intestine. Ng et al. (2018) looked into the possibility that curcumin can aid in IBS manifestation. Crohn’s disease (CD) and ulcerative colitis (UC) are the two primary forms of inflammatory bowel disease (IBD), characterized by abdominal pain, bloating, altered bowel habits, and increased stool frequency. Holt et al. (2005) carried out a pilot study to see how curcumin therapy affected IBD patients who had earlier received standard UC or CD therapy. Curcumin with standard treatment exerts more beneficial effects than placebo plus conventional UC treatment in maintaining recovery, according to Hanai et al. (2006). Bundy et al. (2004) examined that abdominal pain or discomfort score was lowered significantly by 22% and 25% in the one- and two-tablet group volunteers, respectively, and revealed the role of *C. longa* on IBS pathology.

### Dyspepsia and Gastric Ulcer

Six hundred milligrams of curcumin five times a day for 12 weeks to individuals with peptic ulcers could prevent ulcer development but also cause symptomatic erosions, dyspepsia, and gastritis in some patients. Abdominal pain along with other symptoms has greatly decreased with curcumin within 1–2 weeks. Kim et al. (2005) found that orally administered ethanolic *C. longa* extract decreased stomach acid, gastric juice secretion, and ulcer initiation in male rats by inhibiting H2 histamine receptors, which is similar to the effects of ranitidine. Similarly, the antiulcer action of *C. longa* ethanolic extract was seen as it lowers ulcer index in addition to stomach acidity significantly. *C. longa* extract also suppressed hypothermic-restraint stress depletion of stomach wall mucus and diminished the severity of necrotizing agent-induced lesions.

### Antidepressant Properties

Rats suffering from chronic mild stress (CMS) lead to much less sucrose intake; increased IL-6, TNF-α, CRF, and cortisol levels; smaller medulla oblongata; and reduced splenic NK cellular activity. The condition created in CMS was cured with ethanolic extract, which even caused the medulla oblongata to be lower than normal. *C. longa* has antidepressant potential because it tends to hinder monoamine oxidase accumulation in the central nervous system (Yu et al., 2002). Curcumin has a wide range of characteristics that are important to depression pathogenesis. The ethanolic *C. longa* extract prevented the decrease in serotonin, noradrenalin, and dopamine concentrations while increasing serotonin turnover, cortisol levels, and serum corticotrophin-releasing factor levels (Xia et al., 2007). The consequences of orally administered curcumin seem on behavior under chronic stress or depression condition in the rat model. Curcumin administration showed a similar impact to imipramine, a known antidepressant drug, and it has been indicated by various authors to be a feasible alternative source in depression condition (Mohammed et al., 2019; Qi et al., 2020).

### Curcumin Prevents Drug Resistance

Curcumin possesses a powerful anti-drug resistance agent. It has a novel capacity to suppress adriamycin-induced elevation of P-glycoprotein and its mRNA, and this ability is linked to increased intracellular drug accumulation, thereby increasing ADM lethality (Xu et al., 2011). Curcumin blocks NF-*κ*B activation, which results in chemosensitivity in drug-resistant cancer cells. Furthermore, curcumin and tamoxifen co-therapy has also been illustrated to expose tamoxifen-resistant breast cancer cells, suggesting that it could be a viable method for either minimizing tamoxifen resistance or re-sensitizing refractory illness to tamoxifen therapy (Mimeault and Batra, 2011).

### Antimicrobial and Synergistic Property

The essential oil as well as extracts of *C. longa* can suppress a diverse range of bacteria, infectious fungus, and parasites.

### Antibacterial Activity

Some of the researchers assessed the antimicrobial activity of curcumin against different bacterial strains such as *Salmonella paratyphi*, *Trichophytongypseum*, *Staphylococcus aureus*, *Streptococci mutans*, and *Mycobacterium tuberculosis* (Tajbakhsh et al., 2008; Maghsoudi et al., 2017). The extract showed antimicrobial activity towards *Trichophyton longifusus*, *Microsporum canis*, and *S. aureus* while toxicity was indicated against *Lemna minor*. The *C. longa*-treated rabbit group had a significantly greater mean value for wound contraction, and therefore, wounds revealed decreased inflammation and a rising tendency in collagen formation. The extract of *C. longa* in ethanol was active for *Shigella flexneri*, *Staphylococcus epidermis*, *Klebsiella pneumoniae, Lactobacillus*, *Pseudomonas aeruginosa*, *Vibrio cholerae*, and *Salmonella typhi* (Oghenejobo and Bethel, 2017). Drug combinations can cause powerful or reductive pharmacokinetic effects, which may enhance or diminish the clinical efficiency of one another *via* regulation of absorption, distribution, metabolism, and excretion (Chou, 2006). Another study revealed the synergistic combinatorial impact of copper metal ions with aqueous extracts of *C. longa* against *Paenibacillus popilliae*, a known food spoilage bacterium, and detected the phytoconstituents including alkaloid, flavonoid, anthocyanin, steroids, and coumarin in *C. longa* extracts (Jassal et al., 2015). *C. longa* aqueous extract and chitosan possess significant synergism and antibacterial potency at 512 μg/ml and 1,024 μg/ml against MDR pathogens such as methicillin-resistant *S. aureus*, carbapenem-resistant *Pseudomonas*, carbapenem-resistant Enterobacteriaceae and AmpC-producing Enterobacteriaceae*,* and antibiofilm producers (Etemadi et al., 2021). The aqueous extract, curcumin component, and oil fraction of *C. longa* revealed antibacterial activity and suppresses *H. pylori*, *Streptococcus, Staphylococcus,* and *Lactobacillus* species (Mahady et al., 2002).

### Synergistic Interaction

Curcumin has been identified to have synergistic effects but not antagonistic effects when combined with antibiotics such as oxacillin, ampicillin, ciprofloxacin, gentamicin, amikacin, polymyxin B, and norfloxacin, and anti-inflammatory effects when combined with a certain cytotoxic agent, with chemotherapy (Lin et al., 2007), or with a polyphenol derivative-containing diet (Strimpakos and Sharma, 2008). Various researchers have employed different extracts prepared from medicinal plants to treat MDR bacteria (Mehta and Jandaik, 2016; Urmila Jandaik et al., 2016; Mehta et al., 2021; Mehta et al., 2022a), which has been recognized as a global concern. A mixture of *C. longa*, galanga powder, and essential oil of lemongrass slowed the deterioration of raw white hard clam muscle, which improved the seafood quality during preservation (Nguyen, 2020).

### Antifungal Activity

Curcumin was shown to improve the activity of common azole and polyene anti-fungal (Sharma et al., 2010). Another study indicated that *C. longa* oil suppressed dermatophytes and pathogenic fungus when applied externally on guinea pigs infected with dermatophytes, molds, and yeast. The guinea pigs with dermatophytes and fungal infected lesions improved, and the lesions become invisible after 7 days of *C. longa* administration. The antifungal, antibacterial, phytotoxic, cytotoxic, and insecticidal activities of a *C. longa* ethanolic extract were explored by Khattak et al. (2005). Ether, chloroform, and ethanol extracts of *C. longa* along with its oil possess antifungal effect against *Aspergillus flavus*, *Aspergillus parasiticus, Fusarium moniliforme*, and *Penicillium digitatum*, as suggested by several authors (Wuthi-Udomlert et al., 2000; Jayaprakasha et al., 2001). *C. longa* methanolic extract exhibited antifungal action for *Cryptococcus neoformans* and *Candida albicans*, which indicates minimum inhibitory concentration (MIC) values of 128 and 256 g/ml, respectively. Curcumin has antifungal action over all Candida test strains, at MICs varying from 250 to 2,000 g/L, but is less efficacious than fluconazole, according to a recent analysis. It could be attributable to changes in membrane-associated ATPase activity, ergosterol production, or proteinase secretion (Neelofar et al., 2011).

### Antiviral and Antiparasitic Activity

Curcumin has antiviral potential (von Rhein et al., 2016) even for HIV; inhibiting HIV-1 LTR promoter directed gene expression with no effect on cell viability (Ashraf, 2018). Curcumin had moderate effectiveness towards *Plasmodium falciparum* and *Leishmania* organisms. The ethanol extracts exhibit anti-*Entamoeba histolytica* activity while curcumin has anti-*P. falciparum* and anti-*Leishmania* effect *in vitro*. Curcumin seems to have its antiviral activity for Epstein–Barr virus and HIV (Taher et al., 2003). An extract of *C. longa* in both aqueous and ethanol is used in aquaculture as a treatment for bacterial infections (Sahu et al., 2005). Curcumin exerts anti-parasitic action against African trypanosomes, has schistosomicidal activities against *Schistosoma mansoni* adult worms, and has anti-malarial in addition to nematocidal effects. Diets supplemented with *C. longa* reduced small intestine lesion scores and boosted weight gain in chicks infected with the cecal protozoan, *Eimeria maxima*. Curcumin fits well to the active site of the protease, according to *in silico* modeling studies (Vajragupta et al., 2005) and proved to be a powerful inhibitor of HIV integrase, as it can bind acidic residues in the integrase’s catalytic site, limiting it from interacting with its substrates. Molecular docking analysis revealed that particularly the keto-enol and terminal o-hydroxyl group of curcumin are tightly linked to the integrase’s binding region formed by residues such as Glu92, Thr93, Asp116, Ser119, Asp64, His67, Thr66, Asn120, and Lys159 (Vajragupta et al., 2005). Recent analysis has also shown the therapeutic potential of *C. longa* against coronavirus disease 2019 (COVID-19) (Emirik, 2020), and its ability to modulate cytokine storm in COVID-19 patients (Valizadeh et al., 2020; Mehta et al., 2022b) has produced formidable renewed interest in *C. longa.*

### Antifertility

Traditional medicine has been recommended by the World Health Organization as a cost-effective substitute to manufactured antifertility medicines. Parkes mouse strain was given aqueous rhizome extract of *C. longa via* the oral route (600 mg/kg body weight/day for 8 and 12 weeks), which causes reversible spermatogenesis, decreased seminiferous tubules diameter, and loosening of germinal epithelium, thus indicating its potential in male fertility. Hembrom et al. (2015) also examined the influence of an aqueous *C. longa* rhizome extract in sperm count, spermatozoa motility, and seminal pH in Swiss Albino male mice leading to infertility. The combined action of curcumin and andrographolide significantly suppressed the number of implants and litter size in female Sprague–Dawley rats, changed the duration of phases involved in the estrus cycle, and lowered the number of ovarian follicles (Shinde et al., 2015). Petroleum ether in addition to aqueous extract of rhizome shows antifertility impact on rats *via* oral administration and results in complete inhibition of implantation. Curcumin also reduces human sperm motility, suggesting its usage as intravaginal contraceptive and its antispermatogenic activity.

### Immunomodulatory

In the management and therapy of diseases caused by immune system malfunctioning, immune response modulation is necessary. The most commonly used immunosuppressant in transplant rejection is cyclosporin A, a microbial peptide (Elgert, 2009); however, cyclosporin A exhibited toxicities and adverse effects like nephrotoxic activity and gingival hypertrophy. Unfortunately, the majority of commercially available medications include adverse effects. The main important side effects of NSAIDs include injury to the stomach and intestinal mucosa. Corticosteroids, an immunosuppressive medicine, have several negative health risks, including decreased bone marrow and skin fragility. Natural products continue to be a valuable source of innovative and safe anti-inflammatory compounds (Elgert, 2009). Due to the existence of bioactive metabolites, many *Curcuma* species, including *C. longa, C. zanthorrhiza* Roxb.*, C. amada* Roxb.*, C. manga* Valeton & Zijp*, C. aeruginosa* Roxb., and *C. zedoaria* Rosc., have been shown to have a variety of immunomodulatory effects. Several recent analyses on the phytochemistry, biological, and pharmacological action of the *Curcuma* genus have been published (Rajkumari and Sanatombi, 2017; Sun et al., 2017; Mahadevi and Kavitha, 2020; USDA, 2021). Curcumin can inhibit the expansion of T cells triggered by plant lectin concanavalin A (Con A), according to a report on the role of the genus *Curcuma* and its bioactive metabolites to control the immunological response. Curcumin inhibits lymphoma B-cell proliferation by lowering the potency of c-MYC, BCL-XL, and NF-κB. Curcumin has also been demonstrated to suppress the production of ROS in macrophages. Curcumin also stimulates NK cell apoptosis by modulating the NF-κB pathway and inhibiting BCL-XL and Cyclin D. Curcumin inhibits IL-1 and IL-6 inflammatory cytokines such as from LPS-stimulated dendritic cells and suppresses the expressions of CD80, CD86, and MHC class II by dendritic cells. Curcumin also causes reduced LPS-induced MAPK activation and NF-κB p65 translocation in dendritic cells (Nair et al., 2017) along with impaired activation of Th1 responses. Curcumin significantly suppressed the formation of IL-6, IL-8, TNF-α, and MCP-1 from higher glucose-cultured monocytes, according to Jain et al. (2009). Curcumin decreased NOS activity and macrophages’ ability to secrete nitric oxide (NO) at low doses. Curcumin has been revealed to be linked to the viral S1 protein, which is required for SARS-CoV-2 entry in an *in silico* approach; thus, it may inhibit cytokine storm in the severe stage of COVID-19 (Pawitan, 2020). Table 2 shows the immunomodulating activity of curcumin with its mechanisms of action.

**TABLE 2:** Curcumin of *C. longa* with immunomodulating activity and their mechanisms of action.

Main constituent	Subjects	Study design	Immunomodulatory activities	Modulation in parameters/mediators affected	References
Curcumin	Healthy albino mice	*In vivo*	White blood cell production and weight lymphoid organs	Stimulates lymphoid organs and white blood cells	Afolayan et al. (2018)
	Dendritic cells	*In vitro*	Surface molecule expression	Suppresses expression of CD80, CD86, MHC class II, and IL-1	Kim et al. (2005)
	Dendritic cells	*In vitro*	Cytokine production	Decreases production of IL-6, IL-12, and TNF-α	Kim et al. (2005)
	Dendritic cells	*In vitro*	Phosphorylation of mitogen-induced protein kinases (MAPKs) and NF-κB p65 translocation	Inhibition of LPS-induced MAPK activation and nuclear translocation of NF-κB p65	Kim et al. (2005)
	Bronchoalveolar of Balb/c mice	*In vivo*	Allergic response	Decreases number of eosinophils	Ravikumar and Kavitha (2020)
	Bronchoalveolar of Balb/c mice	*In vivo*	Cytokine production	Decreases level of IL-4	Ravikumar and Kavitha (2020)
	PBMCs	*In vitro*	T-cell proliferation	Inhibit the proliferation of lymphocyte	Yadav et al. (2005)
	PBMCs	*In vitro*	Cytokine production	Inhibits the production of IL-2 and TNF-α	Yadav et al. (2005)
	PBMCs	*In vitro*	NF-κB	Inhibit lipopolysaccharide-induced NF-κB	Yadav et al. (2005)
	Erythroleukemic cell line K562	*In vitro*	Cytotoxicity	Increases NK cell cytotoxicity	Yadav et al. (2005)
	Lupus BALB/c mice	*In vivo*	Adaptive immune response	Decreases the percentage of Th1, Th2, and Th17	Kalim et al. (2017)
	Lupus BALB/c mice	*In vivo*	Antinuclear antibody (ANA) levels	Decreases level of ANA	Kalim et al. (2017)
	Monocytes and liver macrophages	*In vivo*	ROS production	Increased the production of ROS	Inzaugarat et al. (2017)
	Monocytes and CD4^+^ cells	*In vivo*	TNF-α and IFN-γ production	Enhanced the production of TNF-α in monocytes and IFN-γ in CD4^+^ cells	Inzaugarat et al. (2017)
	Fish	*In vivo*	Immune response	Increased the expression of antimicrobial peptides	Alambra et al. (2012)

## Nano-Formulations and Green Synthesis of *C. longa* and Its Related Compounds

In the last few years, the use of nanoformulation antibiotics has become increasingly popular in improving pharmacological therapeutic benefits. Nanomedicine is a relatively new field that is rapidly expanding in conjunction with nanotechnology and pharmaceutical disciplines (Caster et al., 2017). Nanopharmaceuticals, on the other hand, confront numerous hurdles, including improved characterization, toxicity issues, regulatory standards, high expenses, and healthcare warnings. Curcumin’s practical applications are frequently limited by factors such as poor water solubility and physicochemical instability, poor bioactive absorption, rapid metabolization, low pharmacokinetics, bioavailability, penetration and targeting efficacy, sensitivity to metal ions, alkaline conditions, heat, and light, among others (Flora et al., 2013). These barriers are being overcome by encapsulating curcumin in nanoformulations (nano curcumin) (Yallapu et al., 2012). Integrating curcumin into nanocarriers is an effective and efficient way to improve curcumin’s biological features, such as bioavailability and solubility, long-term circulation, and long-term retention in the body, as well as overcome curcumin’s physiological challenges (Das et al., 2010; Li et al., 2013; Fonseca-Santos et al., 2016). It can also limit unintentional toxicity to neighboring normal cells/tissues by dispersing the intended tissues. Curcumin encapsulated in poly(lactic-co-glycolic acid) nanoparticles showed a ninefold increase in nano curcumin when compared to natural curcumin (Shaikh et al., 2009). Experiments demonstrate that nanoforms of curcumin are effective in the treatment of liver and heart problems (Shimatsu et al., 2012), cancer (Mohanty and Sahoo, 2010), and brain tumors (Mohanty and Sahoo, 2010; Lim et al., 2011). Moreover, curcumin nanoformulation exhibited threefold higher anti-HIV activity in comparison with its free form; obstructed the HIV-1-triggered expression of interleukin-1β (IL-1β), Topo II α, and cyclooxygenase 2 (COX-2); and blocked the synthesis of viral complementary DNA (cDNA) (Gandapu et al., 2011).

**FIGURE 8:**
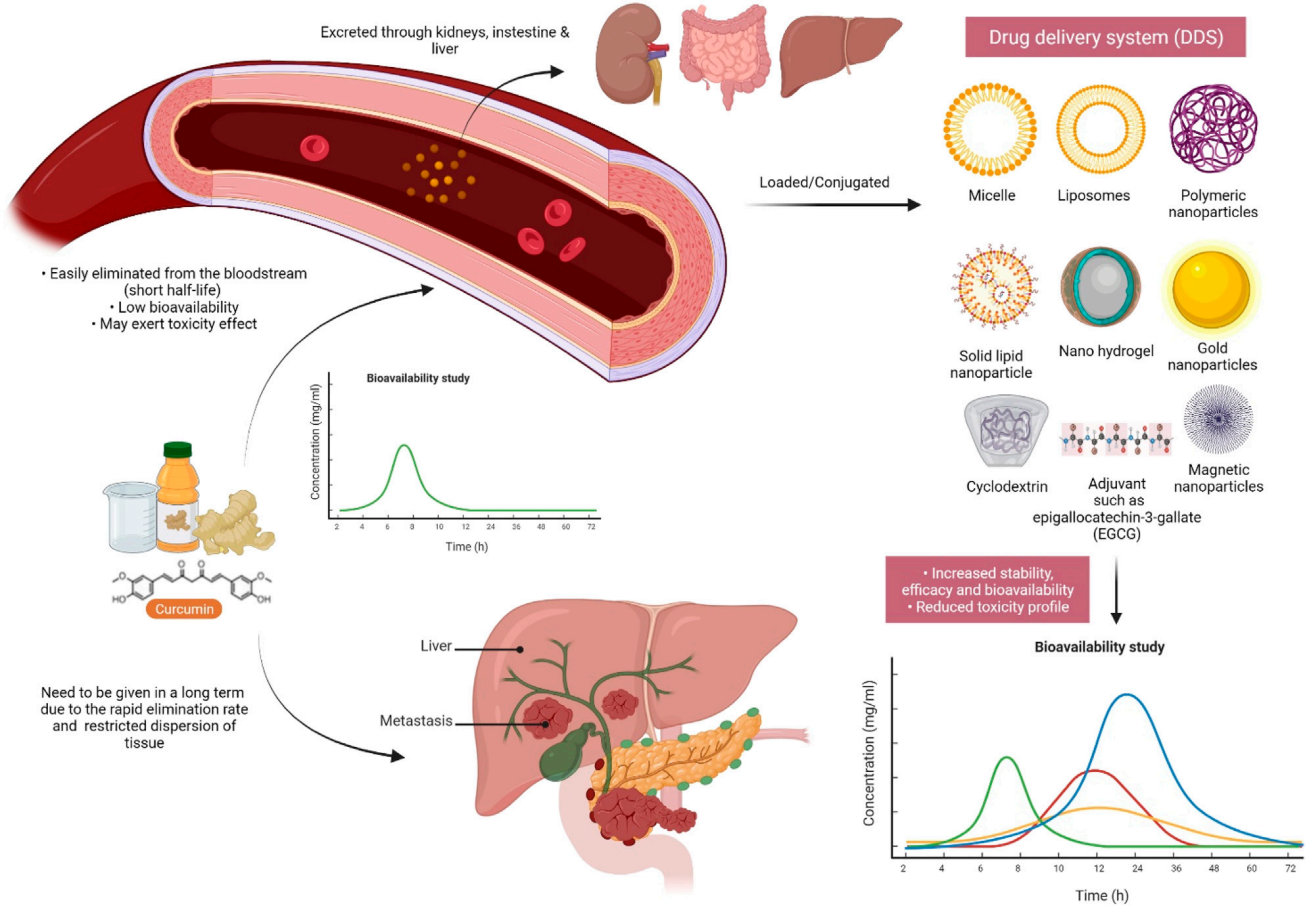
Curcumin must be modified/improved prior to future research due to its extremely low bioavailability. Due to the quick elimination of curcumin in the systemic circulation as a result of enzyme metabolism, conjugating curcumin to adjuvants or other delivery systems may be beneficial in order to increase its half-life and bioavailability.

Moreover, metal-based green synthesis is gaining importance due to its chemical, optical, photochemical, and electronic properties (Mohanpuria et al., 2008). Among several metals, silver has gained huge attention for the green synthesis of NPs because of its numerous applications in various industries, particularly because of its nonlinear optical, biolabeling, and antibacterial capacity. Silver nanoparticles (AgNPs) are widely used in various fields like in drug delivery (Basu et al., 2018), nanomedicine (Carabineiro, 2017), agriculture, and cosmetics, and most importantly, they are used as an antimicrobial agent (Zhang et al., 2017). However, many scientists report that AgNPs also cause toxicity (McGillicuddy et al., 2017), but still, they play a major role as a disinfectant and as a antimicrobial agent. An emergence of nanotechnology that helps in the production of AgNPs has served as a new therapeutic modality. Because of their characteristic broad-spectrum antimicrobial ability, AgNPs have gained increasing attention in biomedical applications including wound management (Parveen et al., 2018; Ahsan and Farooq, 2019; Ravindran et al., 2019), but the hydrophobic nature of curcumin limits its biomedical applications. Hydrogels are in natural or synthetic forms including bacterial cellulose as one of the promising synthetic candidates used in wound dressings because of its ability to maintain a moist microclimate at the wound site, which has been proven to facilitate healing (Tao et al., 2019; Xue et al., 2019). The hydrophobicity of curcumin was overcome by its microencapsulation in cyclodextrins. A combination of AgNP with curcumin in the biosynthetic bacterial cellulose is used for the preparation of hydrogel dressings that exhibit antimicrobial activity against wound-infecting pathogenic microbes like *S. aureus, P. aeruginosa*, and *Candida auris* (Gupta et al., 2020). Among several different approaches to synthesizing AgNP, the use of extracts from natural plant sources has received wide research consideration because of the safe and eco-friendly procedure (Alsammarraie et al., 2018; Hemmati et al., 2019; Ravindran et al., 2019; Keshari et al., 2020). The antibacterial action of nano formed curcumin against a wide range of microorganisms, including fungi, bacteria, and viruses, has been carried out by researchers. Curcumin-modified AgNPs, for example, are utilized to inhibit respiratory syncytial virus (RSV) infection cells and reduce viral loads while having no deleterious side effects (Yang et al., 2016). Naseri et al. then investigated the antiviral effects of curcumin nanomicelles on the attachment and entrance of hepatitis C virus (HCV) infection, finding out that the viral load in HCV cells treated with curcumin nanomicelles was reduced (Naseri et al., 2017). Nanocurcumin was shown to exhibit improved antibacterial activity compared to curcumin because of its enhanced aqueous-phase solubility and simple dispersibility. An efficient antibacterial potential was found against *Bacillus subtilis*, *H. pylori*, *S. aureus,* and *P. aeruginosa* (Basniwal et al., 2011). An aqueous extract from *C. longa-*coated cotton fabrics, as well as formulated metallic AgNPs, also exhibited noticeable antimicrobial potency against *S. aureus*, *P. aeruginosa, S. pyogenes*, and *C. albicans*, and even AgNP-loaded cotton fabrics displayed potent wound healing activity in fibroblast (L929) cells (Maghimaa and Alharbi, 2020). Moreover, recently, it has been found that the combination of AgNP with the rhizome extract of *C. longa* showed antimicrobial effect towards plant affecting bacteria such as *Xanthomonas axonopodis* and *Erwinia amylovora*, which could be useful for nano-drug delivery applications (Gaurav, 2021). Curcumin’s bioavailability was also discussed in Figure 8, as well as a method for dealing with it.

### Side Effects, Contraindications, Precautions, and Safety Aspects of *C. longa*

**FIGURE 9:**
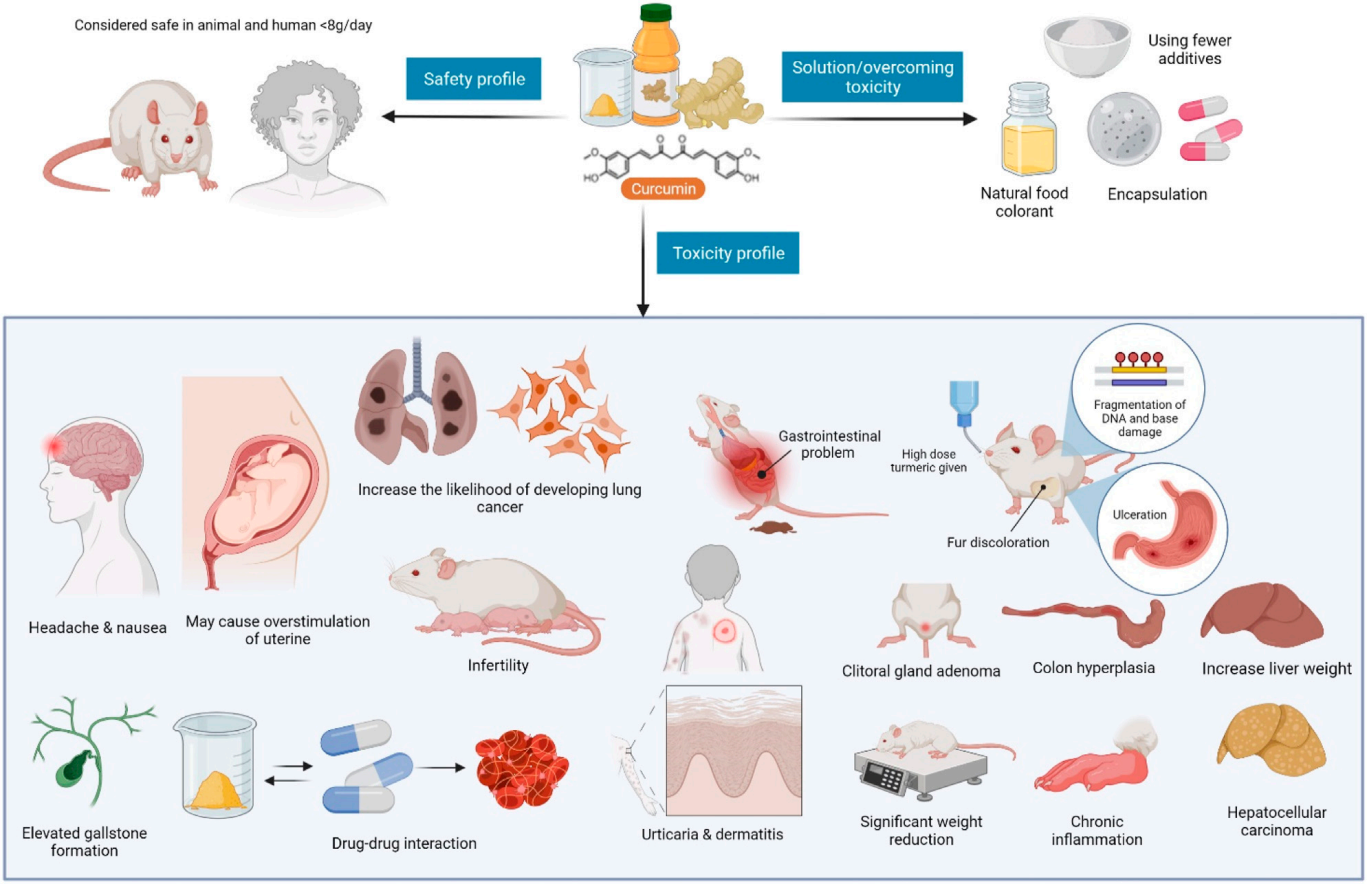
Curcumin safety, toxicity profiles, and solution to overcome it. Curcumin safety, toxicity profiles, and a way to circumvent it. Despite its recognized safety, certain investigations have highlighted unprecedented side effects of curcumin, including gastrointestinal problems, chronic inflammations, and others. To address this issue, fewer additives should be utilized, and curcumin microencapsulation should be tailored.

Facts suggest that excessive turmeric consumption may trigger uterine contraction in pregnancy, and may hinder iron absorption (and so must be used with caution in iron-deficient individuals). Turmeric has been reported to reduce testosterone levels and sperm movement in men (when administered orally) and delay blood clotting (and so its use must be terminated at least 2 weeks before a scheduled surgery). According to some reports, turmeric should not be consumed if one has gallbladder and bleeding problems (Deshpande et al., 1998; Park et al., 2000). Figure 9 depicts curcumin’s safety and toxicity profiles, and a way to manage them (Cheng et al., 2001; Perkins et al., 2002; Rithaporn et al., 2003; Liddle et al., 2006; Sharma et al., 2007; Vareed et al., 2008; Dance-Barnes et al., 2009). In addition, Table 3 summarizes the clinical uses of *C. longa* and its major component curcumin.

**TABLE 3:** Clinical uses of *C. longa* and its major compound curcumin (https://clinicaltrials.gov/).

S. No.	Clinical use	Condition	Intervention	Status
1.	Curcumin as nutraceutical in patients of depression	Major depressive disorder	Dietary Supplement: Curcumin Drug: Fluoxetine	Completed
2.	Premedication with curcumin on post-endodontic pain	Acute pulpitis	Drug: Curcumin	Completed
			Dietary Supplement: 400 mg starch	
3.	Curcumin during the off treatment periods in patients with prostate cancer undergoing intermittent androgen deprivation therapy	Prostate cancer	Dietary Supplement: Curcumin and placebo	Completed
4.	Curcumin and function in older adults	Older adults, physical function, and cognitive function	Drug: Curcumin or microcrystalline cellulose	Completed
5.	Turmeric on new onset primary dysmenorrhea	Primary dysmenorrhea	Drug: Naproxen	Completed
			Dietary Supplement: Turmeric	
6.	Turmeric and exercise-induced muscle damage and oxinflammation	Muscular injury	Dietary Supplement: Turmeric strength for joint and placebo	Completed
7.	Topical application of commercially available *Curcuma longa* gel on superoxide dismutase and malondialdehyde levels in saliva of chronic periodontitis patients	Chronic periodontitis	Drug: Topical application of Curenext gel	Completed
			Dietary Supplement: Placebo	
8.	Turmeric on oxidative modulation in ESRD (end-stage renal disease) patients	End-stage renal failure	Drug: Turmeric	Completed
			Dietary Supplement: Placebo	
9.	Turmeric in gingival massaging and adjunct to scaling and root planing in chronic periodontitis patient	Chronic periodontitis	Procedure: Tooth brushing with dentifrice, turmeric massaging, scaling, and root planing with turmeric massaging	Completed
10.	Turmeric and turmeric-containing tablets and sebum production	Skin inflammation	Dietary Supplement: Turmeric or turmeric-containing combination tablet or placebo tablets	Completed

## Conclusion

*C. longa* with its various pharmacological features has been characterized as a universal panacea among herbal remedies, as per the literature survey. This plant is regarded as a potent medicinal plant with a wide range of potent pharmacological properties due to the presence of numerous chemical components including starch, essential elements, proteins, vitamins, volatile oils, curcumin, and curcuminoids. Curcumin has a long history of use as a culinary spice and food color, as well as a component of Ayurvedic and Chinese medicine. Curcumin has a variety of beneficial effects on humans, according to science. Curcumin is still utilized as a cooking ingredient today, but modern technology has made it possible to use it in a range of food- and health-related applications. Curcumin’s efficacy, safety, and pharmacokinetics have all been examined extensively in clinical studies over the last 50 years (Gupta et al., 2013; Subramani et al., 2018). The development of innovative nanomedicine formulations to increase curcumin targeting, pharmacokinetics, efficacy, and cellular uptake has been prompted by a significant therapeutic limitation (Salehi et al., 2020a; Salehi et al., 2020b). Cancer, CVD, arthritis, atherosclerosis, diabetes, gastric illness, IBD, psoriasis, acquired immunodeficiency syndrome, and other inflammatory disorders are all examples of pleiotropic activities. Several studies in this review discovered the anti-inflammatory effects of *C. longa* and curcumin, including decreased white blood cell, neutrophil, and eosinophil numbers, as well as protective effects on serum levels of inflammatory mediators like phospholipase A2 and total protein in various inflammatory disorders. Curcumin has anticancer properties by interfering with many cellular systems and inhibiting/inducing the production of multiple cytokines, enzymes, or IκKβ, TNF-α, STAT3, COX-2, PKD1, NF-κB, epidermal growth factor, and MAPK, among others. Under oxidative stress conditions, *C. longa* extracts and curcumin decreased MDA and NO levels while increasing thiol, SOD, and catalase levels. Curcumin also influenced the lifespan of organisms by regulating important signaling pathways such as the mTOR, PKA, and FOXO signaling pathways. In conditions where the immune system was disturbed, treatment with *C. longa* and curcumin enhanced IgE, IL-4, TGF-β, IL-17, IFN-γ, and the Th1/Th2 ratio. The pharmacological effects of *C. longa* extracts and curcumin on respiratory, allergy, and immunologic problems suggest that *C. longa* and curcumin may have a possible therapeutic effect on these illnesses. *C. longa* extracts and curcumin delay the onset of diabetes, improve β-cell functioning, prevent β-cell death, and reduce insulin resistance in animal models. Curcumin’s use has been limited due to its low water solubility, which can result in poor chemical stability, oral bioavailability, and cellular uptake. Other strategies that have been aggressively studied include delivering medications at a controlled rate, slow delivery, and targeted delivery. Curcumin nanoformulations have been produced to improve the solubility and bioavailability of the compound. Curcumin’s medicinal applications and clinical efficacy could be expanded if biotechnology and nanotechnology were used to address the current limitations.

**FIGURE 10:**
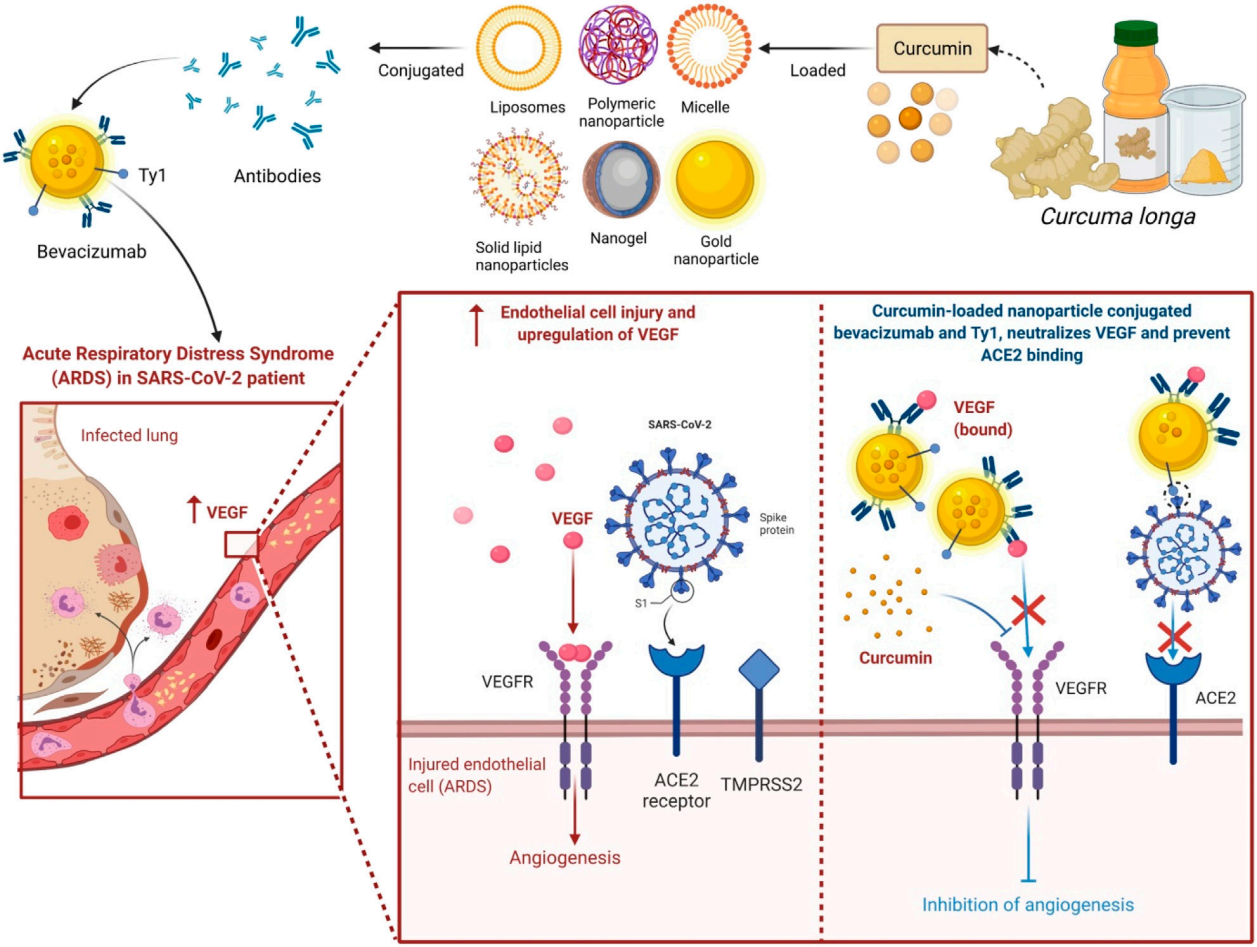
Future perspective of curcumin-loaded gold nanoparticles functionalized with Ty1 and bevacizumab in a patient infected with SARS-CoV-2 (COVID-19).

This review adds to the growing body of evidence supporting the use of turmeric as a preventative and therapeutic strategy. We believe that more progress in the development of strategies incorporating natural products can be exploited to be used against COVID-19 in the upcoming years. Hence, this paper also suggests the use of gold nanoparticles in combination with neutralizing antibody Ty1, which may assist selectively in the receptor binding domain of the SARS-CoV-2 spike, directly hindering angiotensin-converting enzyme 2 interaction. The inclusion of curcumin and bevacizumab further enhanced the efficacy of the proposed strategy as both may target and potently neutralize VEGF, thereby decreasing and slowing tumor growth (Figure 10).

## Future Directions

*C. longa* entailed extensive research and development work to fully exploit its medicinal value, and efforts should be made to investigate the possibilities of practical clinical applications as well as the details of hidden and untold areas in order to maximize its utility for the benefit of humanity. It is suggested to inhibit cytokine storm in the severe stage of COVID-19. So, we can further work on it to get out of this pandemic situation of COVID-19 mutations. For the rational usage of turmeric and curcumin in the therapies of human diseases especially COVID-19, an accurate knowledge of effective dose, safety, and mode of action is necessary.
